# Insights on the Structural and Metabolic Resistance of Potato (*Solanum tuberosum*) Cultivars to Tuber Black Dot (*Colletotrichum coccodes*)

**DOI:** 10.3389/fpls.2020.01287

**Published:** 2020-08-20

**Authors:** Josep Massana-Codina, Sylvain Schnee, Pierre-Marie Allard, Adriano Rutz, Julien Boccard, Emilie Michellod, Marilyn Cléroux, Stéphanie Schürch, Katia Gindro, Jean-Luc Wolfender

**Affiliations:** ^1^Plant Protection Research Division, Agroscope, Nyon, Switzerland; ^2^School of Pharmaceutical Sciences, University of Geneva, Geneva, Switzerland; ^3^Institute of Pharmaceutical Sciences of Western Switzerland, University of Geneva, Geneva, Switzerland; ^4^Changins College for Viticulture and Enology, University Western Switzerland, Nyon, Switzerland

**Keywords:** quantitative resistance, metabolomics, *Solanum tuberosum*, black dot (*Colletotrichum coccodes*), hydroxycinnamic acid amide, steroid derivative, phellem

## Abstract

Black dot is a blemish disease of potato tubers caused by the phytopathogenic fungus *Colletotrichum coccodes*. Qualitative resistance (monogenic) that leads to the hypersensitive response has not been reported against black dot, but commercial potato cultivars show different susceptibility levels to the disease, indicating that quantitative resistance (polygenic) mechanisms against this pathogen exist. Cytological studies are essential to decipher pathogen colonization of the plant tissue, and untargeted metabolomics has been shown effective in highlighting resistance-related metabolites in quantitative resistance. In this study, we used five commercial potato cultivars with different susceptibility levels to black dot, and studied the structural and biochemical aspects that correlate with resistance to black dot using cytological and untargeted metabolomics methods. The cytological approach using semithin sections of potato tuber periderm revealed that *C. coccodes* colonizes the tuber periderm, but does not penetrate in cortical cells. Furthermore, skin thickness did not correlate with disease susceptibility, indicating that other factors influence quantitative resistance to black dot. Furthermore, suberin amounts did not correlate with black dot severity, and suberin composition was similar between the five potato cultivars studied. On the other hand, the untargeted metabolomics approach allowed highlighting biomarkers of infection, as well as constitutive and induced resistance-related metabolites. Hydroxycinnamic acids, hydroxycinnamic acid amides and steroidal saponins were found to be biomarkers of resistance under control conditions, while hydroxycoumarins were found to be specifically induced in the resistant cultivars. Notably, some of these biomarkers showed antifungal activity *in vitro* against *C. coccodes*. Altogether, our results show that quantitative resistance of potatoes to black dot involves structural and biochemical mechanisms, including the production of specialized metabolites with antifungal properties.

## Introduction

*Colletotrichum coccodes* (Wallr.) S. Hughes (Wallroth, 1833; Hughes, 1958) is a ubiquitous phytopathogenic fungus with multiple host plants, including weeds and several crops. It is the responsible for anthracnose in peppers, tomatoes, and onions, and causes black dot in potatoes (*Solanum tuberosum* L.). Several Vegetative Compatibility Groups (VCGs) with different morphology and aggressiveness have been isolated from potatoes, indicating a high genetic variability of the fungal species (Nitzan et al., 2002; Nitzan et al., 2006; Aqeel et al., 2008). Black dot symptoms can be observed in all parts of the plant, and are characterized by the presence of microsclerotia on infected tissue (Read and Hide, 1988). Microsclerotia can survive in the soil for long periods, and high soil inoculum levels result in high disease incidence (Lees et al., 2010). In the field, fungal colonization of roots is followed by colonization of stems, stolons, and tubers (Andrivon et al., 1998). Black dot can affect the yield of potato production (Tsror (Lahkim) et al., 1999), and contamination of tubers with *C. coccodes* results in lesions on the skin of potato tubers and water losses during storage (Lees and Hilton, 2003). Qualitative resistance to black dot has not been reported in potatoes, but different susceptibility levels have been observed among commercially available potato cultivars (Read, 1991; Tsror (Lahkim) et al., 1999; Brierley et al., 2015). Based on disease surveys, it has been suggested that thick-skin cultivars are more resistant to black dot than thin-skin cultivars (Hunger and McIntyre, 1979), and early-maturing cultivars may be less susceptible to the disease because they spend less time in contact with the soil inoculum (Andrivon et al., 1998). Nonetheless, the genetic, morphologic, physiologic, and metabolic basis of host resistance to black dot are still poorly understood. The pressure to produce high quality potatoes, together with the limited efficiency of fungicides in controlling black dot, force developing new strategies to reduce the risk of this blemish disease. Among these, the use of existing cultivars with resistance to black dot is of interest, because it does not require chemical fungicide application.

Plant-pathogen interactions have been studied using different model organisms, including several *Solanaceae* plants. Plants’ response to pathogen attack localizes to individual cells that are in contact with the pathogen and systemic signals from the infection sites (Jones and Dangl, 2006). This response results in important changes on the attacked cell, at transcriptomic, proteomic, and metabolomics levels. Upon pathogen perception, the host cell activates signaling pathways that i) trigger the transcription of defense genes and ii) mediate ROS production and hormone synthesis (Corwin and Kliebenstein, 2017). Ultimately, the plant cell responds to the pathogen attack through defense mechanisms, which involve the synthesis of compounds and metabolic reprogramming (Saijo et al., 2018). Plant resistance to a pathogen is often provided by genes that code for proteins involved in the recognition of pathogens, in the so-called gene-for-gene plant resistance that leads to programmed cell death (PCD) in the hypersensitive response (HR), avoiding the spread of the pathogen (Jones and Dangl, 2006). The plant resistance that leads to the HR is also called qualitative resistance, because pathogen growth is averted and relies on a single gene. In potatoes, several *R* genes have been identified and introduced in breeding programs. As found in many other crops, *R* genes against late blight (caused by *Phytophthora infestans*) contain a nucleotide binding domain (often NB-LRRs) that recognizes the pathogen and triggers an immune response, such as in the case of *R1* or *R2* (Ballvora et al., 2002; Aguilera-Galvez et al., 2018). However, pathogens evolve to the presence of *R* genes in order to overcome resistance (Aguilera-Galvez et al., 2018), and therefore, the presence of a single *R* gene is not sufficient for long-term resistance. On the other hand, quantitative resistance is characterized by limited pathogen growth and symptom development, and often involves multiple plant defense reactions and genes with small to medium effects (Kröner et al., 2011; Corwin and Kliebenstein, 2017). Quantitative resistance is still poorly understood but might involve different mechanisms and is more durable than qualitative resistance.

Plant response to pathogen attack often involves the generation of metabolites that may act as physical barriers, possess antimicrobial activity, or act as signaling molecules (Desender et al., 2007; Kröner et al., 2012). Interestingly, such metabolites can be present before infection, induced by the pathogen, or both (La Camera et al., 2004; Kröner et al., 2012), suggesting that quantitative resistance may be explained by both constitutive and inducible resistance-related metabolites. Cytological (Thangavel et al., 2016), transcriptomic (Zuluaga et al., 2015) and metabolomics studies (Yogendra et al., 2015) of currently available cultivars might help decipher strategies for the resistance of certain cultivars to plant diseases.

Potato tubers are protected from the outer environment through the potato skin, which contains high amounts of proteins involved in the defense response, including enzymes involved in phenolic acid production and in suberization processes (Barel and Ginzberg, 2008). Fungal infection of potato tubers probably requires the penetration of the pathogen through the suberized phellem tissue. Notably, the number of phellem cells, as well as suberin content, correlate with resistance against common scab, produced by the bacterial pathogen *Streptomyces scabies* (Thangavel et al., 2016). Moreover, the penetration of the fungal hyphae in host cells likely produces non-enzymatic reactions, such as oxidative bursts that will impact the biochemistry of the infected cell (Lehmann et al., 2015). Several metabolomics studies have studied the resistance of cultivars and wild *Solanum* species to a number of pests, highlighting biomarkers of resistance (Aliferis and Jabaji, 2012; Pushpa et al., 2014; Yogendra et al., 2015; Chen et al., 2019). Among the defense-related metabolites present in the potato skin, steroidal glycoalkaloids, and calystegines have shown antimicrobial properties (Fewell and Roddick, 1993; Friedman and Levin, 2009), but are also toxic to human consumption, and thus remain in low quantities in commercial cultivars (Petersson et al., 2013; Mariot et al., 2016). On the other hand, the phenylpropanoid pathway leading to the production of various phenolic compounds is activated in potatoes upon microbial infection, and phenolic acids such as the hydroxycinnamic acids (HCA) chlorogenic acid, neochlorogenic acid, and cryptochlorogenic acid have been involved in the resistance of tubers against different diseases (Kröner et al., 2012; Yogendra et al., 2014). Furthermore, flavonoid glycosides, such as rutin and nicotiflorin, are important for determining resistance against several plant pathogens in a number of plants, including potatoes (Bollina et al., 2010; El Hadrami et al., 2011; Henriquez et al., 2012; Kröner et al., 2012; Pushpa et al., 2014). Resistance-related metabolites to late blight have also been identified in potato tubers, and include glucosinolate derivatives, coumarins, and organic acids (Hamzehzarghani et al., 2016).

Metabolomics and cytological analysis have been used to highlight pathogenesis related metabolites in several host-pathogen interactions, including interactions between *S. tuberosum* and microbial pathogens. Although recent studies showed the importance of the host variety and pathogen strain on quantitative resistance (Ors et al., 2018; Samain et al., 2019), studies of plant-pathogen interactions often rely on the use of a model using a single susceptible and a single resistant genotype. Furthermore, the effect of the inoculation of *C. coccodes* on the metabolome of potato tubers has not been studied so far.

In this context, the aim of the present study was to investigate the parameters that influence cultivar resistance to black dot both from a cytological and biochemical perspectives. For this, we use five cultivars with a range of quantitative resistance to black dot in a greenhouse experiment under controlled conditions. All cultivars are either mock or fungal inoculated, allowing the comparison of untreated and inoculated samples. A combined cytological and biochemical approach is used to decipher the mechanisms of resistance. Microscopic analyses of the tuber peel of potatoes with different resistance to plant pathogens have been previously used to highlight structural defense responses in the potato tuber (Thangavel et al., 2014; Thangavel et al., 2016). Here, we combine microscopic observations with a biochemical quantification of the total suberin amount and the composition of this polymer to study the structural differences among potato cultivars and its influence on the resistance to black dot. On the other hand, untargeted metabolomics has been previously used to highlight resistance-related compounds in potatoes against various diseases (Pushpa et al., 2014; Yogendra et al., 2014). Here, we apply an untargeted metabolomics approach (Zhou et al., 2019) combined with multiblock multivariate data analysis (Boccard and Rudaz, 2016) for highlighting features related to the different experimental factors involved. The results are visualized in molecular networks that allow clustering structurally related metabolites with similar fragmentation patterns (Watrous et al., 2012). Furthermore, metabolite annotation is performed using experimental data (www.gnps.ucsd.edu), an *in silico* mass spectral database (Allard et al., 2016) and taxonomical scoring (Rutz et al., 2019) that allow high confidence feature annotation.

The cytological studies and the metabolomics analysis reported here could provide insights on the quantitative resistance of potato tubers to black dot, and highlight biomarkers of resistance to this disease.

## Materials and Methods

### Plant Material

Five cultivars of *Solanum tuberosum* L. commonly grown as table potato cultivars in Switzerland were used for all experiments: Cheyenne (a late-maturing cultivar with red skin), Erika (an early-maturing cultivar with yellow skin), Gwenne (a mid-maturing cultivar with yellow skin), Lady Christl (an early-maturing cultivar with yellow skin) and Lady Felicia (an early-maturing cultivar with yellow skin). Twenty plants of each cultivar were grown for four weeks in sterile conditions as described by Lê (1999). After 24 h of adaptation to non-sterile conditions in a high humidity environment, potato plants were transferred to 4 liters square pots containing an autoclaved substrate consisting of brown and blond peat (Gebr. Brill substrate, Georgsdorf, Germany). Half of the population was *mock*-inoculated (with sterile water) and half of the population was inoculated with *Colletotrichum coccodes*. For fungal inoculations, 10 ml of a conidial suspension (7.5 × 10^5^ conidia/ml) containing mycelial fragments of *Colletotrichum coccodes* (strains 456 and 93 from Agroscope, Nyon, Switzerland) in sterile water was sprayed to the root system during the transfer to pots. Potato plants were grown in a greenhouse for 4 months (from March to July), under a 12-h photoperiod, 20°C to 22°C and 40% to 60% relative humidity, with weekly watering. Following haulm destruction, tubers were kept in the soil for a further month until harvest. For microscopy analysis, naturally infected field-grown tubers from a field trial carried out in Changins (46°23’52.9”N 6°14’19.4”E) in 2016 were used. Tubers were planted in rows of 25 plants each, each row separated by 75 cm, and each plant separated by 33 cm. Conventional agronomic practices were used during plant growth.

### Severity Determination

Daughter tubers from the greenhouse experiment were washed and incubated for 2 weeks under high humidity conditions to allow sporulation of fungi prior to severity determination. 30 single tubers were individually observed under a binocular for the presence of microsclerotia of *Colletotrichum coccodes*. Each tuber was then classified in one of the following classes, depending on the affected area of the tuber: 0 (absence of the fungus), 1 (less than 15%), 2 (between 15% and 35%), 3 (between 36% and 65%) and 4 (more than 65%). The number of tubers of each class was multiplied by the median affected area of the class and used to calculate the average affected area (severity).

### Microscopy Analysis

Tuber peel samples from naturally infected field-grown tubers of all cultivars were harvested and prepared for microscopic analysis after being washed and incubated for 2 weeks under high humidity conditions to allow sporulation of fungi. A black dot symptomatic area and an area free of disease symptoms were selected for each cultivar. Tuber peel samples were prepared as previously described (Hall and Hawes, 1991). Briefly, samples were pre-fixed using a paraformaldehyde (2%) glutaraldehyde (3%) solution at pH 7.0 (0.07 M PIPES buffer) for 3 h at room temperature and descending atmospheric pressure. Subsequently, samples were washed three times with PIPES buffer (0.07M, pH 7.0) and post-fixed with 1% OsO_4_ in 0.07M PIPES buffer for 90 min. Fixed samples were rinsed twice with 0.07M PIPES buffer and stored at 4°C until dehydration and infiltration with the EMbed 812 resin (Electron Microscopy Sciences, Hatfield, PA, USA). Prior to the infiltration with the resin, samples were dehydrated by incubating the samples on growing concentrations of ethanol (30–50–70–95–100% ethanol) for 10 min and continuous agitation in the tissue processor Leica EM TP (Leica Microsystems, Heerbrugg, Switzerland). The ethanol was then replaced by propylene oxide for 30 min and continuous agitation, and the propylene oxide subsequently replaced by the EMbed 812 resin overnight and gentle agitation. To ensure infiltration of the resin, samples were incubated for 2 h under vacuum (400 bars). Polymerization of the resin took place at 60°C for 48 h. Infiltrated samples were then thin cut and resulting sections stained with a mixture of methylene blue (1%), sodium tetraborate (1%), and azur II (1%). Pictures were taken on a Leica DMLB Fluorescence Microscope (Leica Microsystems, Heerbrugg, Switzerland), and phellem thickness measured using the ProgResCapturePro 2.9.0.1 software.

### Suberin Extraction and Depolymerization

Suberin extraction was performed using an adapted protocol from Schreiber (Schreiber et al., 2005). Briefly, lyophilized tuber peel tissues from *mock*-inoculated plants of the greenhouse experiment were cut in 1-cm^2^ pieces and weighed. Discs were then incubated for 72 h in a solution of 2% pectinase and 2% cellulose in citric buffer (10 mM, pH 3.0 adjusted with KOH) containing 1 mM of sodium azide to prevent microbial contamination. Subsequently, periderm tissues were washed with a solution of borate buffer for 24 h, washed again in deionized water, and dried. Waxes were extracted with chloroform (ratio 1 ml CHCl_3_/5 mg periderm) for 18 h. The supernatant was recovered in a clean glass tube, and the pellet reextracted twice with chloroform. The supernatants were combined, dried, and stored. The suberin contained in the dewaxed periderms was depolymerized using BF_3_/MeOH and incubating at 70°C for 18 h. Subsequently, 10 µg of dotriacontane was added as internal standard. The methanolysate was transferred to a new vial containing 2 ml of saturated NaHCO_3_ in water. The residues were washed twice with chloroform, which was then added to the methanolysate. 2 ml of chloroform were added to allow liquid-liquid extraction. The apolar fraction was transferred to a new vial, and the liquid-liquid extraction repeated twice. The extract was washed with milliQ water, and traces of water were removed with the addition of anhydrous sodium sulfate. The extract was then evaporated and stored until analysis.

### GC-MS Analysis of Suberin Monomers

Suberin extracts were resuspended in 100 µl of internal standard solution (methyl nonanedecanoate, 1-tricosanol, and docontriane at 300 mg/L) before being dried under nitrogen. Subsequently, 50 µl of pyridine and 50 µl of BSTFA + 1% TMCS were added to the dry residues and incubated 30 min at 70°C. The sample was dried again under nitrogen before being diluted with 200 µl of dichloromethane and analyzed by GC-MS. GC-MS analysis were performed on a 7890B gas chromatograph (Agilent Technologies, Santa Clara, CA, USA) coupled to a 7010 triple quadrupole mass spectrometer (Agilent Technologies, Santa Clara, CA, USA), equipped with a PAL autosampler MS-2000 (Bruker, Billerica, MA, USA). A split/splitless injector was used in splitless mode with the injector temperature at 250°C. Separation was performed on a DB-5 capillary column (30 m × 0.25 mm, 0.25 µm, film thickness, Agilent Technologies, Santa Clara, CA, USA) and helium as carrier gas was used at 1.2 ml/min in constant flow rate, GC oven was programmed as follow: 100°C hold 2 min followed by 25°C/min increases up to 200°C, hold 1 min, then increased by 3°C/min up to 280°C and maintained at 280°C for 30 min. MS analysis were carried out with electron impact ionization operating at 70 eV and ion source was set at 230°C. The acquisition was performed in full scan mode, with a scan of 30 to 500 amu. Chromatographic data were analyzed using Masshunter B.08.00 software. The mass spectra were compared with reference spectra from library NIST MS Search 2.2 and derivatized pure standards. Calibration curves were constructed by plotting peak areas versus concentrations of selected standards. The standards used were : mix Supelco 37 for fatty acids, tetracosane, methyl tetracosanoate for alkanoic acids and ω-hydroxy acids, 1-hexacosanol for 1-alkanols, dimethyl tetradecanedioate for α, ω-alkanedioic acids, and ferulic acid methyl ester for cis- and trans-ferulic acid. The standards were derivatized as samples. The concentrations of each compound in the extract were calculated by the corresponding calibration curve. The *m/z* used for the quantitation was for alkanoic acids *m/z* 74, for 1-alkanols and ω-hydroxy acids *m/z* 75, for α, ω-alkanedioic acids *m/z* 98 and, respectively, *m/z* 219, 224, 250, and 250 for methyl caffeate, methyl vanillate, methyl coumarate, and methyl ferulate. All solvents were liquid chromatography grade (Carl Roth, Karlsruhe, Germany). Pyridine, BSTFA + 1% TMCS, methyl nonanedecanoate, 1-tricosanol, docontriane, Tetracosane, methyl tetracosanoate, ferulic acid, dimethyl tetradecanedioate, ethyl vanillate, and Supelco 37 were purchased from Sigma Aldrich (Sigma Aldrich, Steinheim, Germany).

### Extraction of Skin Specialized Metabolites

Mock-inoculated (without black dot symptoms) and *C. coccodes*-inoculated (showing black dot symptoms) tubers from the five different cultivars grown in the greenhouse were used for the untargeted metabolomics approach. Potato skins of single tubers were harvested after severity determination, immediately frozen, and lyophilized (n=10). Approximately 300 mg of dry tissue were extracted with 4 ml HPLC-grade methanol (Fisher Scientific, Hampton, NH, USA) containing 1% acetic acid. After centrifugation for 5 min at 4000 rpm, the supernatant was recovered, and the pellet re-extracted with 4 ml methanol containing 1% acetic acid. After centrifugation for 5 min at 4000 rpm, the supernatants were combined, and the solvents were evaporated at 39 mbar of pressure at 40°C (Genevac, SP Scientific, Ipswich, UK). Each extract was dissolved at 5 mg/ml with a 50% methanol aqueous solution and transferred to a vial for UHPLC-MS/MS analysis.

### UHPLC-HRMS/MS Analysis

Chromatographic separation was performed on a Waters Acquity UPLC system interfaced to a Q-Exactive Focus mass spectrometer (Thermo Scientific, Bremen, Germany), using a heated electrospray ionization (HESI-II) source. Thermo Scientific Xcalibur 3.1 software was used for instrument control. The LC conditions were as follows: column, Waters BEH C18 50 × 2.1 mm, 1.7 μm; mobile phase, (A) water with 0.1% formic acid; (B) acetonitrile with 0.1% formic acid; flow rate, 600 μl·min^−1^; injection volume, 2 μl; gradient, linear gradient of 5–100% B over 7 min and isocratic at 100% B for 1 min. The optimized HESI-II parameters were as follows: source voltage, 3.5 kV (pos); sheath gas flow rate (N_2_), 55 units; auxiliary gas flow rate, 15 units; spare gas flow rate, 3.0; capillary temperature, 350.00°C, S-Lens RF Level, 45. The mass analyzer was calibrated using a mixture of caffeine, methionine–arginine–phenylalanine–alanine–acetate (MRFA), sodium dodecyl sulfate, sodium taurocholate, and Ultramark 1621 in an acetonitrile/methanol/water solution containing 1% formic acid by direct injection. The data-dependent MS/MS events were performed on the three most intense ions detected in full scan MS (Top3 experiment). The MS/MS isolation window width was 1 Da, and the stepped normalized collision energy (NCE) was set to 15, 30, and 45 units. In data-dependent MS/MS experiments, full scans were acquired at a resolution of 35,000 FWHM (at *m/z* 200) and MS/MS scans at 17,500 FWHM both with an automatically determined maximum injection time. After being acquired in a MS/MS scan, parent ions were placed in a dynamic exclusion list for 2.0 s. Quality Control (QC) samples containing a mixture of all samples were injected every ten samples throughout the analysis.

### LC-MS/MS Data Processing

LC-MS/MS data files were analyzed by MzMine 2.36 (Pluskal et al., 2010) after converting the ThermoRAW data files to the open MS format (.mzXML) using the MSConvert software from the ProteoWizard package (Chambers et al., 2012). Briefly, masses were detected (both MS1 and MS2 in a single file) using the centroid mass detector with the noise level set at 1.5E5 for MS1 and at 1.0E0 for MS2. Chromatograms were built using the ADAP algorithm, with the minimum group size of scans set at 5, minimum group intensity threshold at 1.0E5, minimum highest intensity was at 1.0E5 and m/z tolerance at 5.0 ppm. For chromatogram deconvolution, the algorithm used was the wavelets (ADAP). The intensity window S/N was used as S/N estimator with a signal to noise ratio set at 25, a minimum feature height at 10,000, a coefficient area threshold at 100, a peak duration ranges from 0.02 to 0.9 min and the RT wavelet range from 0.02 to 0.05 min. Isotopes were detected using the isotopes peaks grouper with a m/z tolerance of 5.0 ppm, a RT tolerance of 0.02 min (absolute), the maximum charge set at 2 and the representative isotope used was the most intense. Peak alignment was performed using the join aligner method (m/z tolerance at 5 ppm), absolute RT tolerance 0.1 min, weight for m/z at 10 and weight for RT at 10. The peak list was gap-filled with the same RT and m/z range gap filler (m/z tolerance at 5 ppm). The resulting aligned peaklist contained 9321 features in negative mode and 10844 features in positive mode. Only variables that appeared in at least 80% of the samples of a group were retained. Furthermore, all variables that were detected in the blanks and represented more than 1% of the average of the samples were eliminated. Finally, only variables that had less than 30% of variation in the Quality Control samples were retained. The application of these filters yielded a total of 5,086 variables for negative ionization mode and 6,186 variables for ionization positive mode that were subjected to statistical analysis. Only features possessing MS2 spectra were kept to build molecular networks using the peak-list rows filter option from the original peaklist, which yielded 2,717 features in negative ionization mode and 2,943 in positive ionization mode. The resistance-related constitutive (RRC) and resistance-related induced (RRI) scores were calculated as the ratio of the mean of abundance in the resistant cultivars/the mean of abundance in the susceptible cultivars in control and inoculated conditions, respectively (RRC = RM/SM, RRI = RP/SP, where RP, resistant genotype with pathogen inoculation; RM, resistant genotype with mock inoculation; SP, susceptible genotype with pathogen inoculation; SM, susceptible genotype with mock inoculation). The qualitative RRI was calculated as the ratio of the induction in the resistant cultivars/induction in the susceptible cultivars (qRRI = (RP/RM)/(SP/SM)).

### Multivariate Data Analysis (AMOPLS)

Analysis of Variance Multiblock Orthogonal Partial Least Squares (AMOPLS) was computed under the MATLAB^®^ 8 environment (TheMathWorks, Natick, MA, United States). The first step of the method is a partition of the data matrix into a series of additive submatrices, each of which is associated with a specific effect of the experimental design. This follows ANOVA principles by computing average values related to each of the factors levels (cultivar, inoculation, and their interaction). This allows the relative variability of each main effect or interaction term to be evaluated using the sum of squares of the corresponding submatrix. A multiblock OPLS model is then computed for the joint analysis of the collection of submatrices to predict level barycenters of the experimental factors and their combinations. Further interpretation is carried out following the OPLS framework, using specific predictive components that are associated with the different effects of the experimental design, and orthogonal components summarizing unexplained residual variability. Samples groupings can be investigated on the corresponding score plots (tp and to, respectively), while variables’ contributions are analyzed using loading plots (pp and po, respectively). Empirical *p*-values are computed using random permutations of the experimental design to assess the statistical significance of each effect using an effect-to-residuals ratio. A series of 10^4^ random permutations was calculated to validate AMOPLS models and evaluate the statistical significance of each main and interaction effect. The interested reader can refer to the original article describing the AMOPLS method for a detailed description (Boccard and Rudaz, 2016).

### Molecular Networking Parameters

A molecular network (MN) was created with the Feature-Based Molecular Networking (FBMN) workflow (Nothias et al., 2019) on GNPS (Wang et al., 2016) (www.gnps.ucsd.edu). The mass spectrometry data were first processed with MZmine (as described above) and the results were exported to GNPS for FBMN analysis. The precursor ion mass tolerance was set to 0.02 Da and the MS/MS fragment ion tolerance to 0.02 Da. A molecular network was then created where edges were filtered to have a cosine score above 0.7 and more than 6 matched peaks. Further, edges between two nodes were kept in the network if and only if each of the nodes appeared in each other’s respective top 10 most similar nodes. Finally, the maximum size of a molecular family was set to 100, and the lowest scoring edges were removed from molecular families until the molecular family size was below this threshold. The spectra in the network were then searched against GNPS spectral libraries (Horai et al., 2010; Wang et al., 2016). The library spectra were filtered in the same manner as the input data. All matches kept between network spectra and library spectra were required to have a score above 0.7 and at least 6 matched peaks. The DEREPLICATOR was used to annotate MS/MS spectra (Mohimani et al., 2018). The molecular networks were visualized using Cytoscape 3.6 software (Shannon et al., 2003). The GNPS job parameters and resulting data are available at the following addresses (https://gnps.ucsd.edu/ProteoSAFe/status.jsp?task=d3d5ddbec02d4df9a12bd02b258b6dcc and https://gnps.ucsd.edu/ProteoSAFe/status.jsp?task=fc46d070ee5a4d5d8cfda6abbdb533b8).

### Metabolite Annotation

The spectral file (.mgf) and attributes metadata (.clustersummary) obtained after the MN step were annotated using the ISDB-DNP (In Silico DataBase—Dictionary of Natural Products), a metabolite annotation workflow that we previously developed (Allard et al., 2016). Annotation was done using the following parameters: parent mass tolerance 0.005 Da, minimum cosine score 0.2, maximal number of returned candidates: 50. Furthermore, taxonomically informed scoring was applied on the GNPS outputs using *Solanum tuberosum* as species, *Solanum* as genus, and *Solanaceae* as family, returning an attribute table which can be directly loaded in Cytoscape. The taxonomically informed metabolite annotation process has been previously described in detail (Rutz et al., 2019). The scripts are available online (taxo_scorer_user.Rmd) at https://github.com/oolonek/taxo_scorer. The chemical classes of the compounds were described using ClassyFire (http://classyfire.wishartlab.com/) (Djoumbou Feunang et al., 2016).

### *In Vitro* Antifungal Bioassay Against Colletotrichum coccodes

The *in vitro* antifungal activity of some of the highlighted compounds in the metabolomics analysis was tested using the food-poisoning method in 48-well plates. Briefly, 10 µl of a 1 × 10^6^ conidia/ml suspension of *C. coccodes* (fungal strain 456, Agroscope) in water were inoculated in Potato Dextrose Broth (PDB) - Potato Dextrose Agar (PDA) medium (70:30) amended with a range of doses of the following compounds: the glycoalkaloids solanine and chaconine, the saponin protodioscin, the tropane alkaloid calystegine A3, the hydroxycinnamic acid amides feruloyltyramineand kukoamine A, the free polyamines spermine and spermidine, the flavonoid glycosides nicotiflorin and rutin, the coumarins esculetin and scopoletin, and the hydroxycinnamic acid chlorogenic acid. All compounds were solubilized in DMSO (final concentration 3.5%, v/v), except for spermine, spermidine, calystegine A3, and chlorogenic acid, which were soluble in PDB. DMSO (3.5% v/v) in PDB/PDA (70:30) or PDB/PDA (70:30) were used as controls. All compounds were tested at concentrations of 10 to 1000 µM, except for chlorogenic acid, that was tested at concentrations of 150 to 15000 µM. Pictures were taken 7 days after inoculation with a Canon EOS 5D and the growth area calculated using the software ImageJ. Growth inhibition was calculated as the percentage of growth reduction respective to the control (100 − (growth area Xi/growth area control *×* 100).

### Statistical Analysis

Arc sinus transformation was applied to severity data before statistical analysis. One-way ANOVA was applied to severity data, phellem thickness, and suberin amounts, followed by the post-hoc Fisher’s LSD test for multiple pair-wise comparisons. For the *in vitro* antifungal bioassay data, mycelial growth of each treatment was compared to the untreated control using the Student’s T-test.

## Results and Discussion

### Commercial Potato Cultivars Exhibit Different Responses to Black Dot

**Figure 1:**
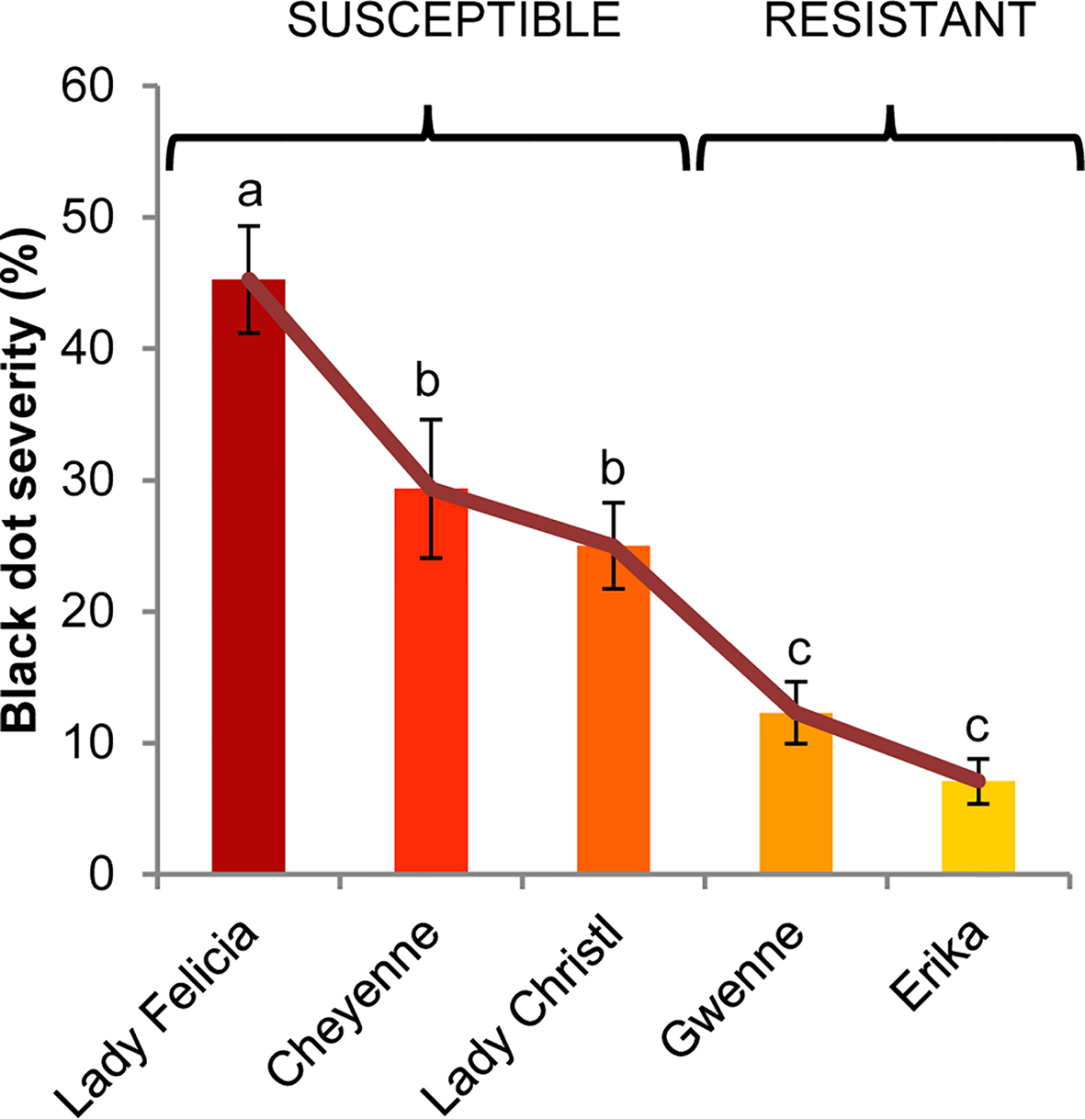
Black dot disease severity of five different potato cultivars artificially inoculated with *C. coccodes*. Disease severity is expressed as the percentage of tuber area showing symptoms of black dot (Means ± Standard Error, n=30). Statistically significant differences are indicated by different letters (p < 0.05, Fisher’s LSD test).

In order to study resistance to black dot, we used five commercially available potato cultivars that had previously shown different resistance levels to black dot in the field. In a greenhouse experiment under control conditions, mock-inoculated plants produced tubers that did not exhibit symptoms of black dot. On the other hand, the percentage of affected tuber area (severity) of black dot in inoculated plants differed among cultivars, with a cultivar exhibiting very high susceptibility (Lady Felicia), two cultivars exhibiting moderate-to-high susceptibility (Cheyenne and Lady Christl), and two cultivars exhibiting low susceptibility (Erika and Gwenne) (**Figure 1**). In particular, these results are comparable to those found in field assays for all cultivars except Cheyenne and Lady Christl, which exhibited lower susceptibility to black dot in the field trials. According to additional field trials (data not shown), overall disease severity was higher in the greenhouse experiment than in field trials, suggesting that the artificial inoculation resulted in a disease pressure higher than that found in the field. It is possible that a higher inoculum than that found in the field was applied to the greenhouse experiment, since soil inoculum correlates with disease severity (Lees et al., 2010) Furthermore, inoculum concentration correlation with disease severity is genotype dependent (Alonso-Villaverde et al., 2011), and both the pathogen strain and the plant genotype influence host resistance in other phytopathogenic interactions (Ors et al., 2018; Samain et al., 2019). These results suggest that a high disease pressure as applied in the greenhouse might negatively influence the resistance of Cheyenne and Lady Christl to black dot. Overall, the cultivars Erika and Gwenne can be considered as resistant to black dot, while Cheyenne, Lady Christl, and Lady Felicia are susceptible to black dot and will be regarded as such along this study (**Figure 1**).

### *Colletotrichum coccodes* Colonizes the Tuber Periderm of the Different Potato Cultivars

**Figure 2:**
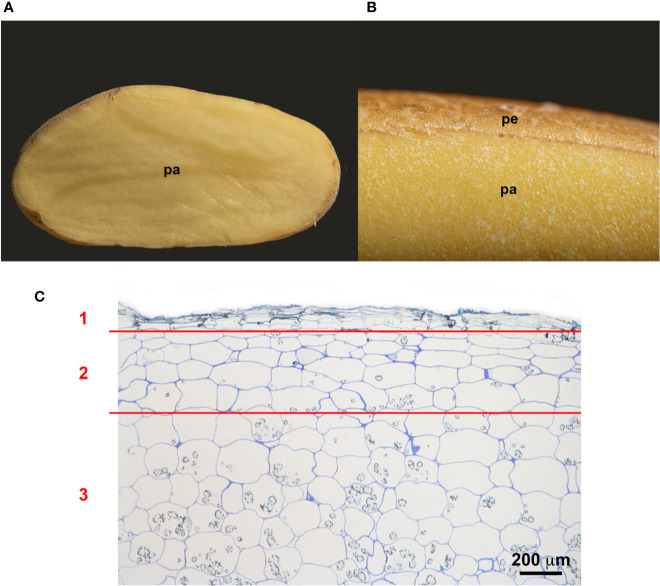
Structure of the potato periderm. **(A)** Longitudinal section of a potato tuber. **(B)** Close view of the potato periderm (pe) and parenchyma (pa). **(C)** Microscopic semithin section of the suberized phellem (1), the starch-depleted phelloderm (2) and the starch-containing storage parenchymal cells (3). Bar corresponds to 200 µm.

**Figure 3:**
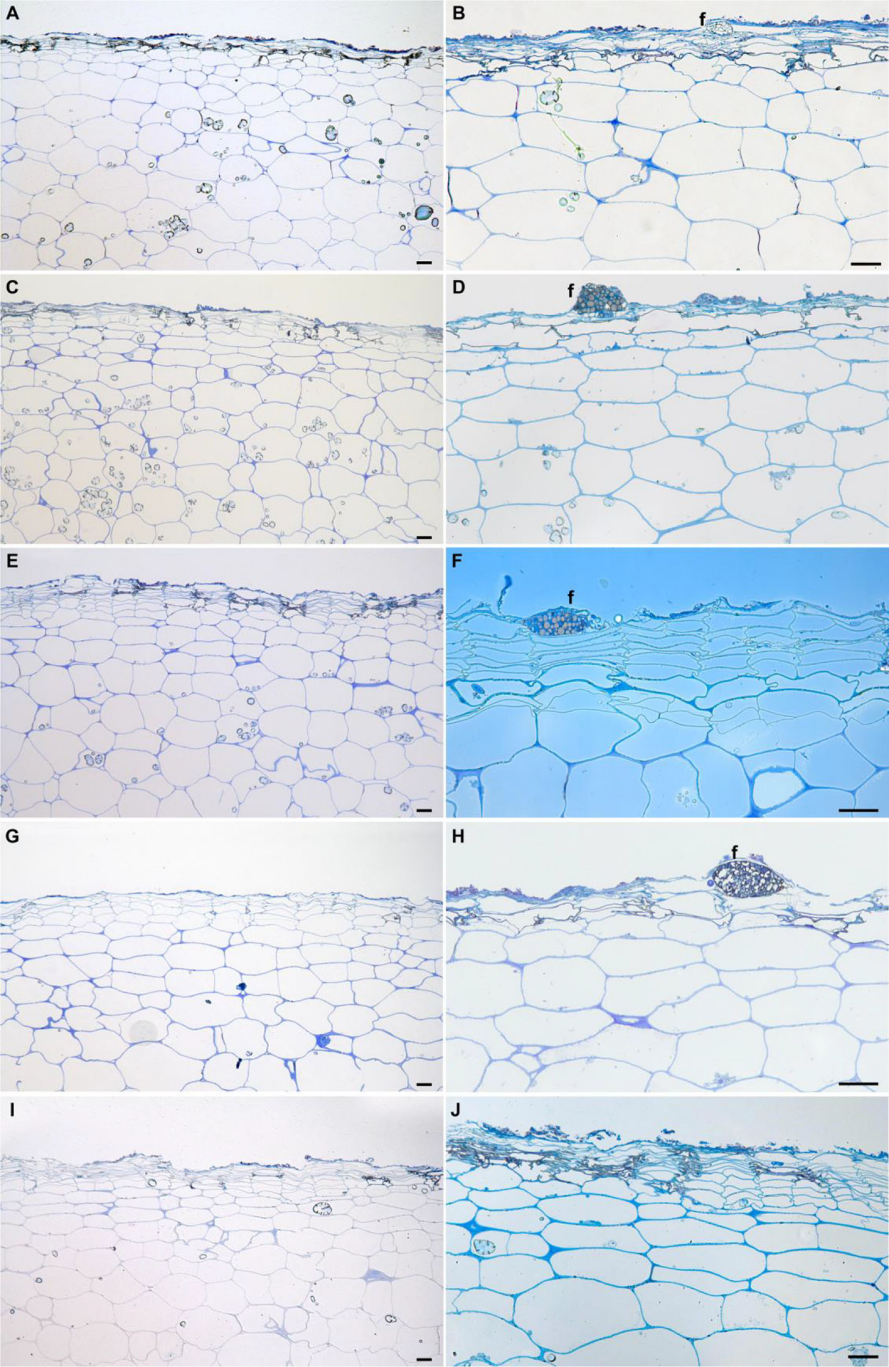
Longitudinal semithin sections of the phelloderm of asymptomatic **(A**, **C**, **E**, **G**, **I)** and black dot symptomatic **(B**, **D**, **F**, **H**, **J)** regions of the field-grown potato cultivars Lady Felicia **(A**, **B)**, Lady Christl **(C**, **D)**, Cheyenne **(E**, **F)**, Gwenne **(G**, **H)**, and Erika **(I**, **J)**. Bars correspond to 50 µm. *f*: fungal structures.

**Figure 4:**
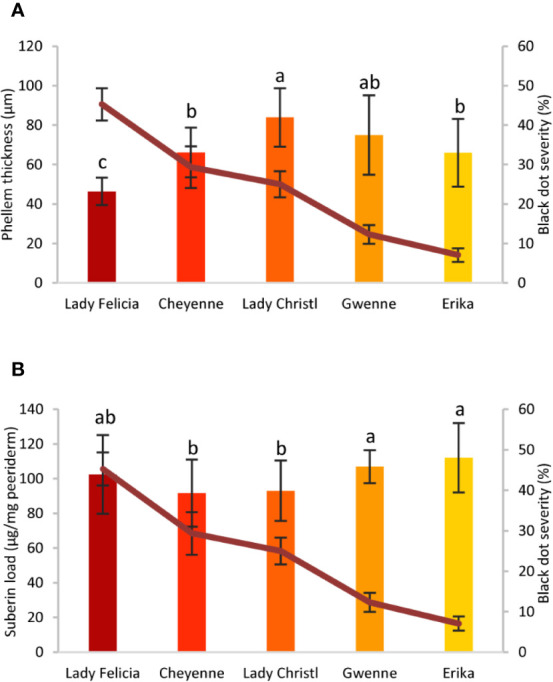
Phellem thickness and suberin concentration of five potato cultivars with different quantitative resistance to black dot. **(A)** Phellem thickness (µm) measured in semithin sections of asymptomatic regions of a potato tuber grown in the field of five cultivars (n=12, mean ± Standard Deviation). **(B)** Total suberin amounts (µg/mg periderm) of five potato cultivars grown under controlled conditions in the absence of the fungal pathogen (n=6). Statistically significant differences in phellem thickness or suberin load are indicated by different letters (p < 0.01, Fisher’s LSD). In all graphs, black dot severity is shown in a red line and its axis on the right.

Potato tubers are storage organs in which the most abundant compartment is the flesh, which contains large amounts of starch granules (**Figures 2A, C**). Tuber flesh is separated from the outer environment by the skin, or periderm, formed by suberized phellem cells and unsuberized phellogen cells (**Figures 2B, C**). Interestingly, *C. coccodes* hyphae were found through the periderm of potato tuber zones showing symptoms of black dot but not in parenchymal tissue, suggesting that *C. coccodes* does not penetrate in these cortical cells (**Figures 3B–J**). Furthermore, regions of potato tubers with black dot symptoms showed a disorganized and collapsed phellem with microsclerotia of *C. coccodes* forming below the surface (**Figures 3B–J**). Notably, collapse of the phellem was observed in all cultivars, although to a lesser extent in the resistant ones since symptoms were less abundant (**Figures 3B–J**). Furthermore, potato cultivars did not respond to *C. coccodes* infection by increasing their periderm layer (**Figure 3**), in opposition to what is observed in response to *Streptomyces scabies* (Khatri et al., 2011). These results suggest that *C. coccodes* induces the collapse of the phellem, which might be responsible for the higher permeability of infected tubers, resulting in water losses during storage. In fungus-free tuber skin regions, the thickness of the phellem ranged from 46 to 84 µm, with between 6 and 10 cellular layers, in the five different cultivars (**Figures 3** and **4A**). Interestingly, phellem thickness was the lowest in the most susceptible cultivar, suggesting that thin-skin cultivars are more susceptible to black dot. That is in accordance with USA surveys, where Russet-type cultivars (thick skin) were more resistant to black dot than thin-skinned cultivars (Hunger and McIntyre, 1979). However, it is worth noting that Russet-type cultivars, which are not commonly used in Europe, possess thicker skins than the cultivars used in our study, with skin thickness of usually more than 150 µm (Artschwager, 1924). Moreover, no correlation between skin thickness and resistance to black dot was found between the other four cultivars, suggesting that other mechanisms are involved in the resistance to black dot.

Phellem cells are surrounded by the polymer suberin, which protects plants from both biotic and abiotic stresses (Vaughn and Lulai, 1991), and potato variants with broad tuber-disease resistance produce higher amounts of suberin (Thangavel et al., 2016). Total suberin concentrations ranged from 92 to 112 µg/mg periderm in the five cultivars studied, values comparable to those found elsewhere (Company-Arumí et al., 2016). Notably, small differences in the total concentration of suberin were observed among cultivars. Total suberin was higher in the two resistant cultivars than the mid-susceptible cultivars, but no significant differences were observed between the resistant and the most susceptible cultivars (**Figure 4B**). Furthermore, the composition of the suberin polymer did not strongly differ among potato cultivars, and clear differences were not observed for any of the suberin monomers (**Supplementary Table S1**). Altogether, these results suggest that thin-skin cultivars such as Lady Felicia are more susceptible to black dot, but that quantitative resistance to black dot cannot be fully explained through the structure of the tuber skin. Furthermore, suberin amount or composition do not explain the differences in the phellem structure and do not correlate with black dot resistance in control conditions. Whether suberin production and accumulation is involved in the reaction of potato tubers to *C. coccodes* infection and induced resistance to black dot remains to be studied.

### Cultivar-Specific Metabolites Are Highlighted Using Untargeted Metabolomics

**Figure 5:**
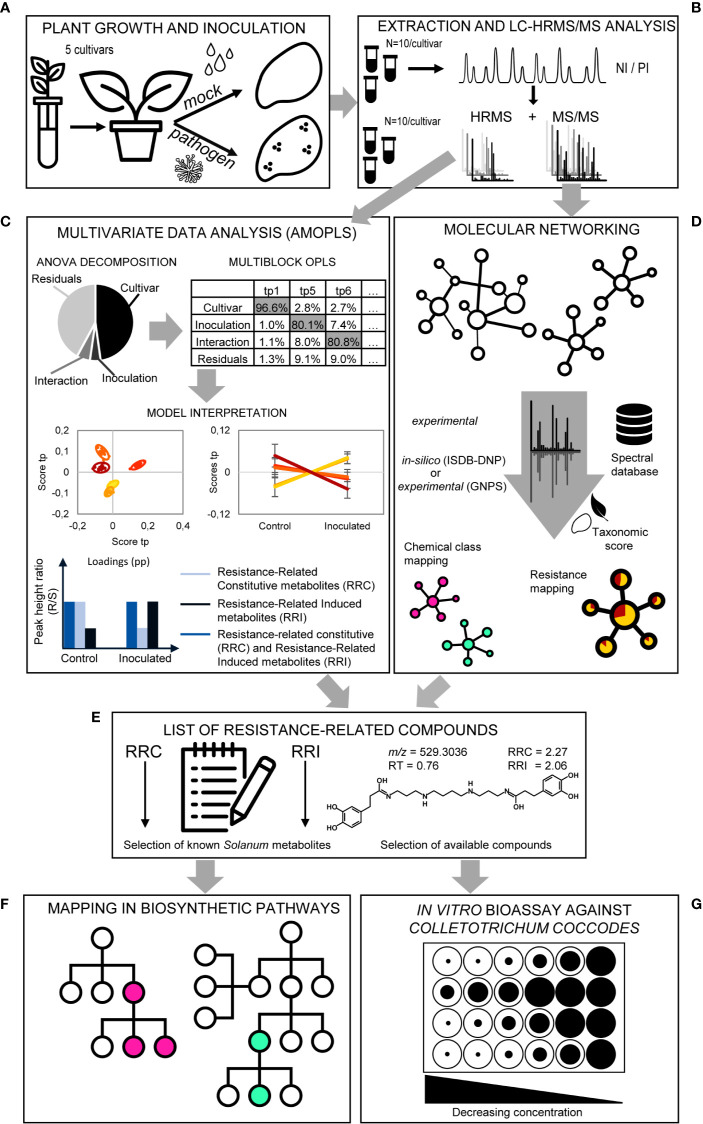
Overview of the metabolomics strategy. **(A)** *Plant growth and inoculation*. Potato plantlets of the five studied cultivars are grown *in vitro* and transferred to the greenhouse in pots using sterilized soil to avoid fungal infection. Half of the population is *mock* inoculated, while the other half is inoculated with *C. coccodes*. **(B)** *Extraction and LC-HRMS/MS analysis*. Methanolic extraction of specialized metabolites is followed by LC-HRMS/MS (data-dependent mode) analysis in both negative ionization (NI) and positive ionization (PI) modes. **(C)** *Multivariate Data Analysis (AMOPLS)*. All features detected in full scan MS are analyzed using an AMOPLS model. ANOVA decomposition of the matrix in submatrices allows assessing the relative variability of each effect (i.e., cultivar, inoculation, interaction, and residuals) by computing the sum of squares of the submatrices. This is followed by a multiblock OPLS model, the predictive components of which explain one of the effects (see **Supplementary Table 2**). AMOPLS separates the sources of variability related to the experimental factors with dedicated predictive score (tp) and loading values (pp) that are used for the model interpretation. Predictive scores (tp) highlight sample groupings (i.e., cultivar, see **Figure 6**) or trends (i.e., upon inoculation, see **Figure 9**) and loadings (pp) highlight induced molecules with respect to the different experimental factors to evidence Resistance-Related metabolites. **(D)** *Molecular Networking*. Features possessing MS/MS spectra are used to build a Molecular Network (MN) based on spectral similarity. Features are then annotated by spectral comparison with experimental (GNPS) or *in silico* (ISDB-DNP) spectral databases. *In silico* annotations are re-ranked using taxonomic proximity information. Color of individual clusters of the MN are set according to the most abundant chemical class found in the cluster (consensus chemical class) or the relative intensity in resistant and susceptible cultivars (see **Figure 7**). **(E)** *List of Resistance-Related compounds*. A combination of the AMOPLS data analysis and the MN visualization is used to build a list of Resistance-Related metabolites, Constitutive (RRC) or Induced (RRI), with their annotations, and sorted according to their RRC or RRI scores (see **Supplementary Table 3**). **(F)** *Mapping in biosynthetic pathways*. A selection of Resistance-Related annotated metabolites previously reported in the *Solanaceae* family are placed in their biosynthetic pathways according to the Kyoto Encyclopedia of Genes and Genomes (KEGG) for *Solanum tuberosum* (or related plant species, if not available for *S. tuberosum*) to highlight putatively induced or repressed biochemical pathways (see **Figure S6**). **(G)** *In vitro* bioassay against *Colletotrichum coccodes*. A selection of highlighted Resistance-Related metabolites, commercially available pure compounds, are tested in an *in vitro* bioassay against *C. coccodes* using the food-poisoning method at decreasing concentrations from 1000 µM to 10 µM (see **Figure 8** and **Figure S5**).

In an attempt to understand the interaction between *S. tuberosum* and *C. coccodes* and, ultimately, decipher quantitative resistance of potatoes to black dot, an untargeted metabolomics approach was carried out on mock and fungal inoculated plants of the five cultivars studied (**Figure 5A**). Specialized metabolites were extracted with a methanolic solution and profiled by UHPLC-HRMS/MS (Zhou et al., 2019) (**Figure 5B**). In order to study the complex and large amount of data obtained, a dual method combining statistical multivariate analysis and molecular networking was used (**Figures 5C, D**). Notably, the full factorial experimental design allowed the study of the genotype effect (cultivar), the effect of the inoculation process, and the interaction of both factors, which can be used to highlight biomarkers. On one hand, the metabolic effects associated with the experimental factors were analyzed in a dedicated supervised statistical model, i.e., AMOPLS, combining ANOVA decomposition of the LC-MS data according to the experimental design, and multiblock orthogonal partial least squares modeling (**Figure 5C**, **Supplementary Table S2**). In addition, metabolite annotation was carried out by a combination of feature based molecular networking (FBMN) (Nothias et al., 2019) and dereplication against experimental data from the GNPS platform (http://gnps.ucsd.edu/) and against an *in-silico* fragmentation database (ISDB) weighted using taxonomical data (**Figure 5D**) (Allard et al., 2016; Rutz et al., 2019). Furthermore, a selection of the annotated compounds was identified by comparison of the HRMS, MS/MS spectra and RT with authentic standards, which allowed the establishment of experimental anchor points and the confident propagation of annotations through the network. Dereplication through the ISDB of the features that possessed a MS/MS spectrum yielded annotations for 69% and 74% in NI and PI mode, respectively. The most represented metabolic classes in the potato tuber skin were lipid and lipid-like molecules (which include fatty acyls, prenol lipids, and steroid derivatives), phenylpropanoids (including cinnamic acids, and flavonoids, among others), organic oxygen compounds, and organoheterocyclic compounds (including benzopyrans, lactones, and indole derivatives) (**Supplementary Figure S1**). In order to highlight specific biomarkers, the AMOPLS results and the FBMN were combined. Concerning the AMOPLS analysis, the ‘cultivar’ main effect was the most important in determining the differences among samples in both PI and NI modes, and represented 48% of the total metabolome variability (**Supplementary Table S2**). These results indicate that the tested commercially available cultivars grown under controlled conditions differ in their biochemical composition, suggesting that this might explain biological or physiological properties. For example, only one of the five cultivars studied has a red skin (Cheyenne), while the other four cultivars have yellow skins. The AMOPLS efficiently highlighted the characteristic features of this cultivar. They were putatively annotated as anthocyanins, the isoflavonoid genistein and the flavanonol dihydrokaempferol, among others (**Supplementary Figure S2**). These features were also easily highlighted in the corresponding MN (**Supplementary Figure S3**). Thus, the approach highlighted chemical markers that are very specific to the red skin cultivar Cheyenne, demonstrating that the chosen methodology is efficient to distinguish the different sources of metabolic variations and highlighting biomarkers. This strategy will be used to highlight resistance-related metabolites against black dot and biomarkers of *C. coccodes* inoculation. The main effect ‘cultivar’ in the AMOPLS model will be used to highlight metabolites that are relatively more abundant in the two resistant cultivars than in the three susceptible cultivars under control conditions (Resistance-Related Constitutive metabolites). The main effect ‘inoculation’ will be used to highlight biomarkers of the fungal infection (Pathogenesis-Related metabolites). Finally, the ‘interaction’ between both main effects will be used to highlight metabolites that are specifically induced in the resistant cultivars upon fungal inoculation (Resistance-Related Induced metabolites). Combining the information from the AMOPLS models and the MNs will result in a list of Resistance-Related (RR) compounds (**Figure 5E**). The biosynthetic pathway of these compounds will be searched to highlight induced biochemical pathways (**Figure 5F**), and the antifungal activity of some of these metabolites will be assessed using an *in vitro* bioassay against *C. coccodes* (**Figure 5G**).

### Black Dot Resistance Is Associated With High Constitutive Levels of Steroidal Saponins, Hydroxycinnamic Acids, and Hydroxycinnamic Acid Amides

**Figure 6:**
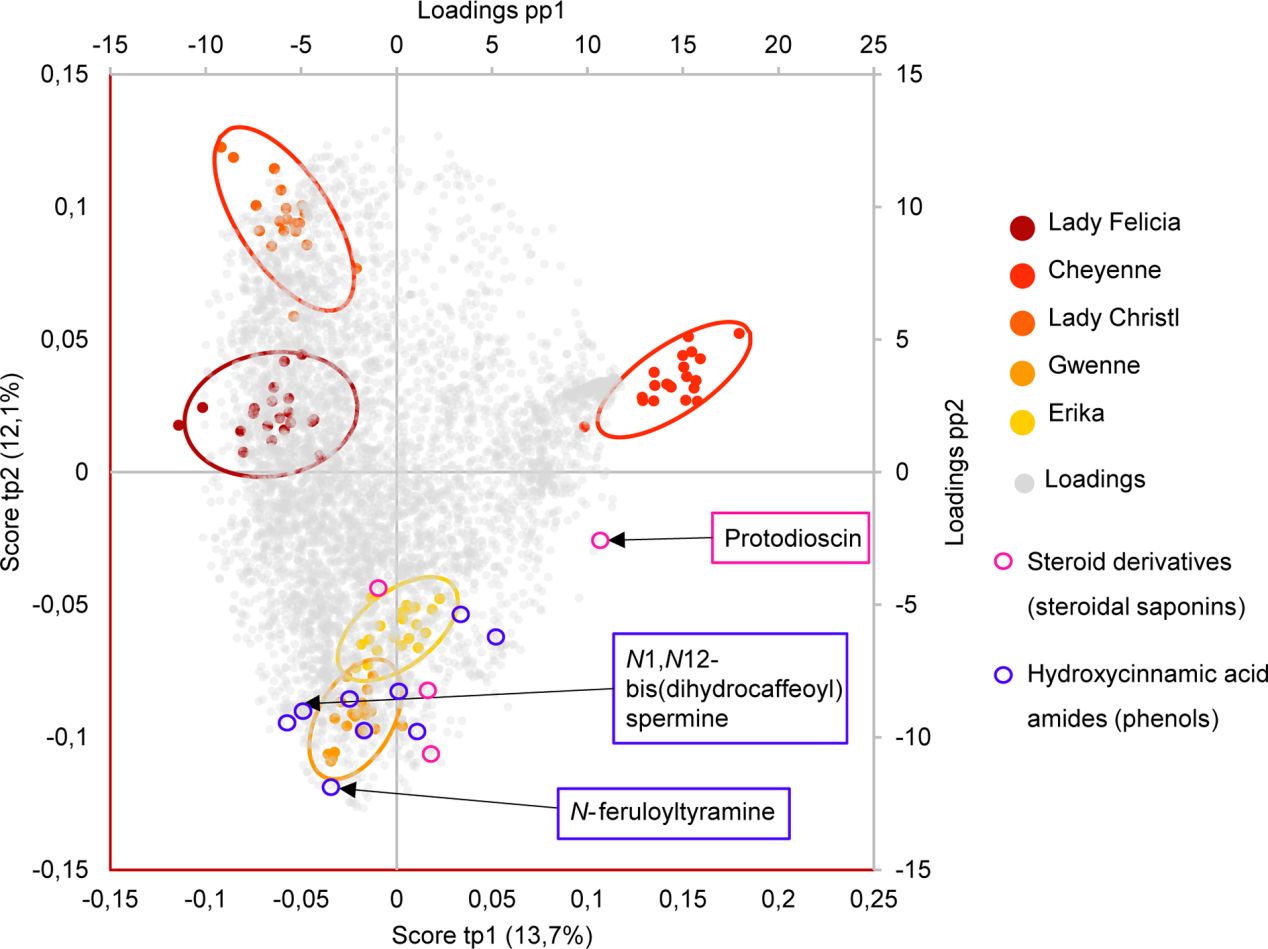
Biplot representation of the two first predictive components of the AMOPLS model in negative ion mode explaining 25,9% of the total variance. Observations are color-coded as cultivars (n=20) with 95% confidence ellipses and positioned according to the left and below axis. Loadings are colored in grey and positioned according to the right and above axis. Resistant cultivars (Erika and Gwenne) have negative scores of tp2, while susceptible cultivars (Lady Felicia, Cheyenne, and Lady Christl) have positive scores of tp2. The loadings of highlighted resistance-related metabolites annotated as hydroxycinnamic acid amides (including *N*-feruloyltyramine and *N*1,*N*12-bis(dihydrocaffeoyl) spermine) and as steroid derivatives (including protodioscin) are indicated in blue and pink, respectively.

**Figure 7:**
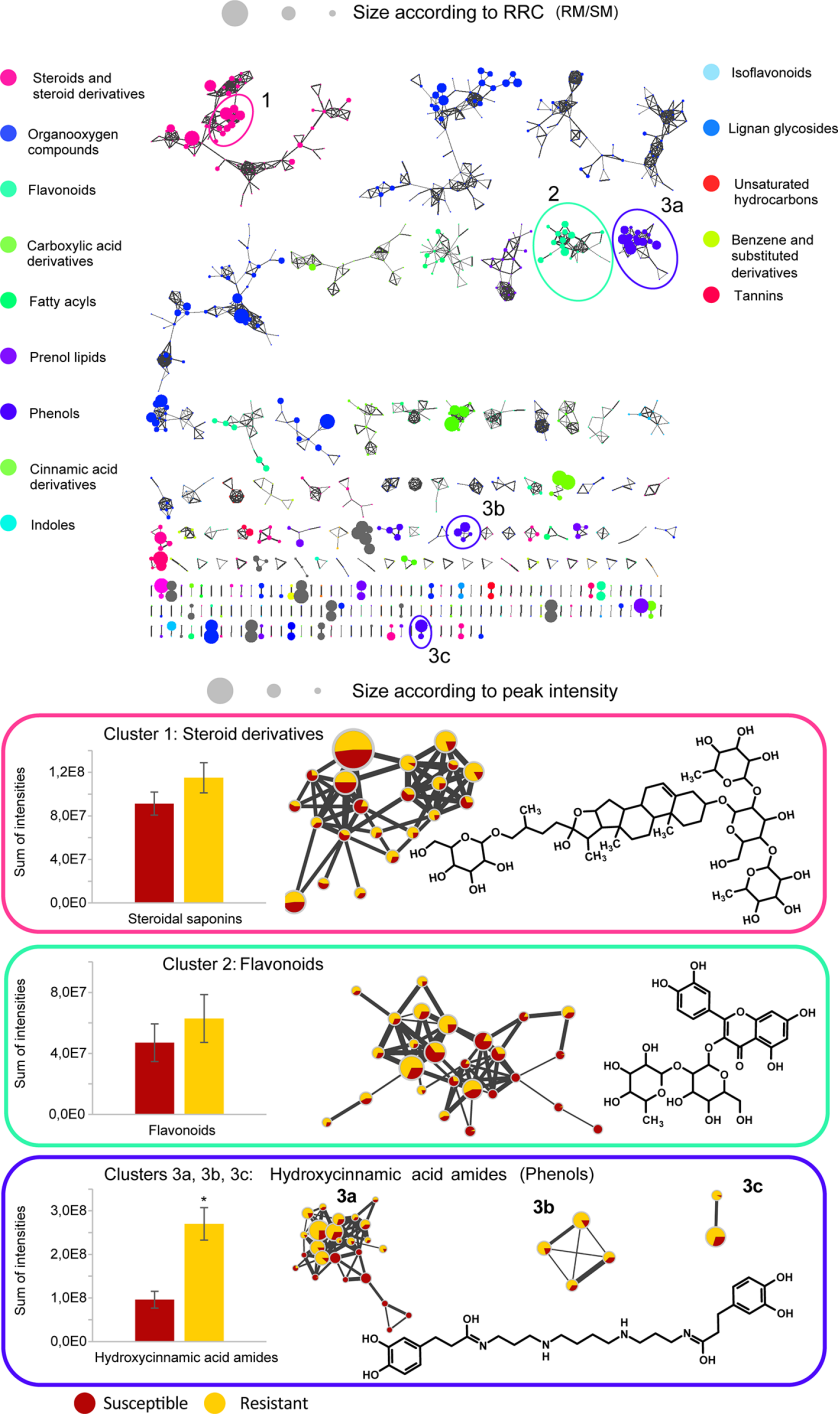
Global Molecular Networks (MNs) in negative ion mode of the five potato cultivars. In the global Molecular Network, color of the node is set according to the consensus chemical class across the cluster, node size is set according to the ratio of intensities between resistant and susceptible cultivars in control conditions (RRC). Molecular families of interest that include compounds confirmed by an analytical standard are highlighted in a cluster, with the structure of the standard displayed, size of the node set according to the average peak intensity (among all samples) and color of the node set as a pie-chart with the relative abundance of the metabolite in the resistant cultivars (yellow) and the susceptible cultivars (red) in control conditions. The average detected intensity (of all features in a cluster) in susceptible and resistant cultivars is shown as a histogram. The average cluster intensity ratio (resistant vs susceptible) provides an estimation of the contribution of the compound class to resistance, the ratio in each node highlights if specific metabolites only are involved. Asterisks indicate significant differences between resistant and susceptible cultivars (p < 0,05, T-test). Only clusters containing at least two nodes are shown, self-loop nodes are not displayed.

**Figure 8:**
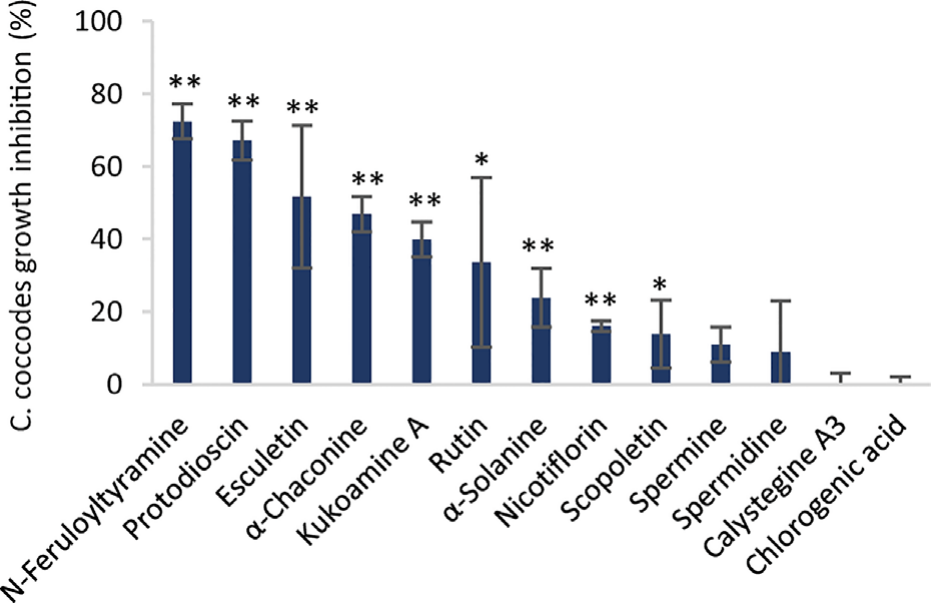
Inhibition of *C. coccodes* growth (%) *in vitro* at 750 µM for chlorogenic acid and at 500 µM for the rest of the compounds (n=3, Mean ± Standard Deviation). Kukoamine A = *N*1,*N*12-bis(dihydrocaffeoyl) spermine. Asterisks indicate significant differences compared to the untreated control (*p < 0,05, **p < 0,01, Student’s T-test).

Differences between the two resistant and the three susceptible cultivars were highlighted by visualizing the second predictive component of the AMOPLS model in both PI and NI modes (**Figure 6**, **Supplementary Figure S2**). Resistance-Related Constitutive (RRC) metabolites are described as those being more abundant in the resistant cultivars than in the susceptible cultivars under control conditions, and the RRC ratio (Resistant Mock/Susceptible Mock) was used to highlight biomarkers in the MN (**Figure 7**). Notably, several clusters of steroid derivatives were evidenced in both PI and NI modes, including the furostanol saponin protodioscin (identified by comparison of MS/MS to a pure standard) and related compounds (cluster 1 in **Figure 7**). Most features in this cluster were putatively identified through comparison with the ISDB to furostanol and spirostanol saponins. In addition, several clusters of lipid and lipid-like molecules, putatively annotated as spirostanol saponins, were also found to be qualitatively more abundant in the resistant cultivars in PI mode (**Supplementary Table 3** and **Supplementary Figure S4**). Notably, these steroidal saponins showed high RRC values and were also highlighted using AMOPLS (**Figure 6**, **Supplementary Figure S2**). Altogether, these results suggest that potato tubers with constitutively high amounts of steroidal saponins, including furostanol and spirostanol saponins, are more resistant to black dot. Steroidal saponins are mainly found in *Liliaceae* and *Agavaceae* plants, but they also have been identified in members of the family *Solanaceae* (Faizal and Geelen, 2013). Their role in plant defense has been proposed as phytoanticipins, because they are produced independently from pathogen detection (Faizal and Geelen, 2013). In order to study whether these compounds might have antifungal activity against *C. coccodes*, an *in vitro* bioassay based on the food-poisoning method was used (**Figure 5G**). Protodioscin was found to strongly inhibit *C. coccodes* growth at 500 µM (**Figure 8**). To the best of our knowledge, steroidal saponins have not been quantified in potato commercial cultivars, but steroidal saponins in wild *Solanum* species are found at concentrations of 100 µM or less (Caruso et al., 2013), a concentration that resulted in 30% growth inhibition of *C. coccodes* (**Supplementary Figure S5**). On the other hand, the most abundant steroidal glycoalkaloids, alpha-chaconine, and alpha-solanine, showed RRC values of 1.40 and 1.16, respectively (**Supplementary Table S3**). These steroidal derivatives, which are found in potatoes and other wild *Solanum* species, have been implicated in plant defense, especially against herbivores (Friedman, 2006). Interestingly, alpha-chaconine has been shown to have higher antifungal activity than alpha-solanine (Fewell and Roddick, 1993), and is usually found at higher concentrations (i.e., 500 µM in the cultivar Snowden) than alpha-solanine (i.e., 250 µM in the cultivar Snowden) in the potato peel (Friedman, 2006). At 500 µM, alpha-chaconine showed higher inhibition of *C. coccodes* mycelial growth than alpha-solanine (**Figure 8**). Furthermore, no significant inhibition was observed by alpha-solanine at 250 µM (**Supplementary Figure S5**). Altogether, these results suggest that high amounts of alpha-chaconine might be fungitoxic to *C. coccodes in planta*. Nevertheless, it is worth noting that the most susceptible cultivar to black dot exhibits higher amounts of these SGAs than the other susceptible cultivars, suggesting that relatively high SGA amounts might contribute to the resistance phenotype, but they are not sufficient *per se*. Alpha-chaconine and alpha-solanine are glycoalkaloids with a solanidine backbone, and, together with the furostanol and spirostanol saponins, derive from squalene (**Supplementary Figure S6**). It is worth noting that brassinosteroids (BRs), which are also steroid derivatives, are plant growth hormones, and that a trade-off between BR signaling and plant defense is often observed (Yu et al., 2018). Indeed, some pathogens overcome plant resistance in potato by inducing the BR pathway (Turnbull et al., 2017). Although BRs were not detected in our untargeted analysis, our results suggest that the balance between plant defense and BR signaling may differ between resistant and susceptible cultivars.

Several phenylpropanoids were found to be more abundant in the resistant cultivars than the susceptible ones in control conditions. This is the case of the hydroxycinnamic acids (HCAs) chlorogenic acid, neochlorogenic acid, and cryptochlorogenic acid, which were ca. 20% more abundant in the resistant cultivars (**Supplementary Table S2**). Interestingly, the dimers of these compounds are even more abundant in the resistant cultivars (40–84%) and suggest that chlorogenic acid, and its isomers and dimers may be associated with quantitative resistance against black dot. Accumulation of HCAs upon pathogen infection has also been described in other plant-pathogen systems, including anthracnose in sweet pepper caused by *C. coccodes* (Mikulič Petkovšek et al., 2009; Mikulic-Petkovsek et al., 2013). Chlorogenic acid is the most abundant HCA in potato tubers (Navarre et al., 2011) and it has been shown to have a direct antimicrobial effect against *Phytophthora infestans* and, especially, *Pectobacterium atrosepticum*, suggesting a stronger activity against bacteria than to fungi-like oomycetes (Kröner et al., 2011; Kröner et al., 2012). In our bioassays, chlorogenic acid did not to inhibit *C. coccodes* growth at concentrations as high as 7.5 mM (**Supplementary Figure S5**), suggesting that this resistance-related metabolite does not possess direct antifungal activity against *C. coccodes in vitro*.

Other clusters highlighted as RRC include a cluster of flavonoid glycosides (cluster 2 in **Figure 7**). Interestingly, flavonoid glycosides more abundant in resistant cultivars included nicotiflorin (RRC of 1.55) and the most abundant flavonoid glycoside rutin (RRC of 1.41), which have been involved in the resistance of potato tubers to soft rot caused by *P. atrosepticum* (Kröner et al., 2012) and, the latter, has shown antifungal activity (Báidez et al., 2007; Pereira et al., 2008). At 500 µM, rutin showed stronger antifungal activity than nicotiflorin (**Figure 8**). However, previous studies have quantified rutin and nicotiflorin at ca. 100 and 50 µM in the potato skin, respectively (Kröner et al., 2012), concentrations that did not inhibit the growth of *C. coccodes in vitro* (**Supplementary Figure S5**). It is worth noting that some of these flavonoid glycosides were abundant in the most resistant (Erika) cultivar but not in the second most resistant one (Gwenne) (**Supplementary Table S3**), suggesting that flavonoid glycosides might be involved in the resistance of potato tubers against black dot especially in the cultivar Erika.

Interestingly, a cluster of compounds highlighted as abundant in the resistant cultivars included *N*1,*N*12-bis(dihydrocaffeoyl) spermine (kukoamine A) (identified by comparison of MS/MS to a pure standard) (cluster 3a in **Figure 7**), which has been previously identified in potato tubers (Parr et al., 2005). This cluster also includes other features putatively annotated as dihydrocaffeoyl spermines and dihydrocaffeoyl spermidines. Furthermore, another cluster with features putatively annotated as caffeoyl, dihydrocaffeoyl spermines, and spermidines was highlighted as an RRC cluster (cluster 3b in **Figure 7**). Notably, spermine derivatives were abundant in one of the resistant cultivars (Gwenne), and spermidine derivatives were most abundant in the other resistant cultivar (Erika) (**Supplementary Table S3**). On the other hand, the most substituted spermine and spermidine derivatives were found to be more abundant in the susceptible cultivars (**Supplementary Table S3**). Altogether, these results suggest that cultivars resistant to black dot accumulate spermine (Erika) or spermidine (Gwenne) derivatives, except the most substituted forms. Free spermine has been suggested to play a role in the hypersensitive response of tobacco to Tobacco Mosaic Virus (TMV) (Walters, 2003), but free polyamines were not unambiguously detected in our analysis. Furthermore, no antifungal activity against *C. coccodes* was recorded for these two polyamines at concentrations as high as 1 mM. Notably, *N-*feruloyloctopamine and *N-*feruloyltyramine, other hydroxycinnamic acid amides (HCAAs), were also found to be more abundant in the resistant cultivars (cluster 3c in **Figure 7**, **Supplementary Table S3**). The role of HCAAs in plant-pathogen interactions has been studied in several models, such as in *Arabidopsis thaliana*, where they accumulate upon inoculation with *Alternaria brassicicola*, and are required for the defense response against this pathogen (Muroi et al., 2009). HCAAs are synthesized in the cytoplasm and translocated to the plasma membrane through glutathione S-transferases, possibly upon pathogen infection, accumulating in methanol soluble granules in the inner face of the cell wall (Macoy et al., 2015). Peroxidase polymerization of HCAAs in the cell wall results in the integration of HCAAs in the suberin polymer reinforcing the cell wall and providing resistance against pathogen infection (Macoy et al., 2015). HCAAs have also been found to accumulate in potato tubers upon fungal inoculation (Clarke, 1982; Keller et al., 1996), and have been highlighted as resistance-related compounds against late blight (Yogendra et al., 2014). Our results suggest that constitutively enhanced HCAA amounts are a biomarker of black dot resistance in potato tubers. Polyphenolic amides have been found to be more abundant in the potato cultivar Russet Burbank than in other cultivars (Huang et al., 2017), and Russet-type cultivars are more resistant to black dot than thin-skinned cultivars (Hunger and McIntyre, 1979), reinforcing the hypothesis that HCAAs might be involved in the resistance to black dot. Interestingly, the HCAA production rate is higher than its incorporation in the cell wall (Macoy et al., 2015). Since the metabolomics analysis was carried on non-polymerized specialized metabolites, it can be assumed that HCAAs detected are found in the cytosol or in the cell wall before polymerization, indicating that they play a role independently of suberin in the resistance to black dot. Furthermore, suberin levels in non-inoculated samples did not differ among the five cultivars studied, suggesting that constitutively high amounts of HCAAs, but not constitutively high amounts of suberin, correlate with black dot resistance. Notably, *N*1,*N*12-bis(dihydrocaffeoyl) spermine and, especially *N*-feruloyltyramine, were found to strongly inhibit *C. coccodes* growth *in vitro* at 500 µM (**Figure 8**). Moreover, both compounds showed antifungal activity at 50 µM (**Supplementary Figure S5**), the estimated concentration of *N*1,*N*12-bis(dihydrocaffeoyl) spermine in potato tubers (Parr et al., 2005) and *N*-feruloyltyramine in healing potato tuber discs (Negrel et al., 1996). Altogether, our results show that non-polymerized HCAAs are relatively more abundant in potato cultivars resistant to black dot, suggesting that free HCAAs, probably in the inner face of the cell wall, play a role in the defense against *C. coccodes* infection.

### Higher Hydroxycinnamic Acid and Hydroxycoumarin Levels and Lower Levels of Flavonoid Glycosides Are Observed in *C. coccodes* Inoculated Potato Tubers

The ANOVA decomposition step of the multivariate data analysis (i.e., AMOPLS) showed that the inoculation of *C. coccodes* had a statistically significant impact on the potato skin metabolome. The main effect ‘inoculation’ was found to be responsible of 5% of the total variability observed in the analysis (**Supplementary Table 2**). One single component of the AMOPLS models in both PI and NI modes explained the contribution of the fungal inoculation to the total variability (**Supplementary Table S2**), and the loadings of this component were used to detect metabolites exhibiting intensity fold changes upon fungal inoculation. Several phenylpropanoids, including features putatively annotated as HCAs (feruloylquinic acid) and HCAAs (*N*-caffeoylputrescine and *N*-dihydrocaffeoylputrescine), were relatively more abundant upon *C. coccodes* inoculation. Interestingly, even the most abundant HCA (chlorogenic acid) and its dimer showed a significant positive intensity fold-change in inoculated samples (**Supplementary Table S3**). This is in line with other studies that have shown that total phenolics increase in potato leaves upon bacterial infection (Poiatti et al., 2009) and in potato tubers upon inoculation of the oomycete pathogen *P. infestans* (Kröner et al., 2011; Kröner et al., 2012). Moreover, an important increase was observed in *N-*dihydrocaffeoylputrescine and *N*-caffeoylputrescine, but not in other HCAAs. Putrescine HCAAs have been shown to accumulate in rust-infected wheat (Samborski and Rohringer, 1970) and in *Nicotiana tabacum* cells upon inoculation of *P. syringae* (Baker et al., 2005). Altogether these results suggest that HCA and putrescine derivative production are enhanced in potato tubers infected by *C. coccodes* during growth (**Supplementary Figure S6**). A group of hydroxycoumarins, including scopoletin (identified by comparison of MS/MS to a pure standard), isofraxidin, and dimethylfraxetin showed a positive intensity fold-change upon inoculation (**Supplementary Figure S7A**). Coumarins have been shown to accumulate upon pathogen attack in several plant species, especially in *Arabidopsis thaliana* and in *Nicotiana tabacum* (Stringlis et al., 2019) and, in potatoes, they have been detected in tubers infected with *Phoma exigua* var. *foveata* (Malmberg and Theander, 1980). Our results suggest that hydroxycoumarins accumulate in potato tubers upon *C. coccodes* infection.

It was noted that different features exhibited lower intensity in potatoes after inoculation, suggesting a degradation or downmodulation of the corresponding metabolites upon fungal inoculation. Quercetin and kaempferol glycosides were found in lower ratios in inoculated samples of all cultivars except for the red-skin cultivar Cheyenne (**Supplementary Table 3**). Nonetheless, kaempferol glycosides were more abundant in Cheyenne than in the rest of the cultivars, making the visualization of the decrease in kaempferol derivatives less obvious (**Supplementary Table S3**, **Supplementary Figure S7B**). Interestingly, quercetin-3-O-rutinoside, but not kaempferol-3-O-rutinoside, has been shown to possess antimicrobial activity against plant pathogens (El Hadrami et al., 2011; Kröner et al., 2012). Altogether, these results suggest a direct or indirect degradation of flavonoid glycosides by *C. coccodes*. This could in turn limit the antimicrobial properties of the potato tuber skin. It is worth noting that coumaric acid is the precursor of all metabolites affected by the inoculation of *C. coccodes* (HCAs, HCAAs, coumarins, and flavonoids), and the different pathways involving coumaric acid may be affected during pathogen infection (**Supplementary Figure S6**).

### Hydroxycoumarins and Steroid Derivatives Are Specifically Induced Upon *C. coccodes* Inoculation in Resistant Cultivars

**Figure 9:**
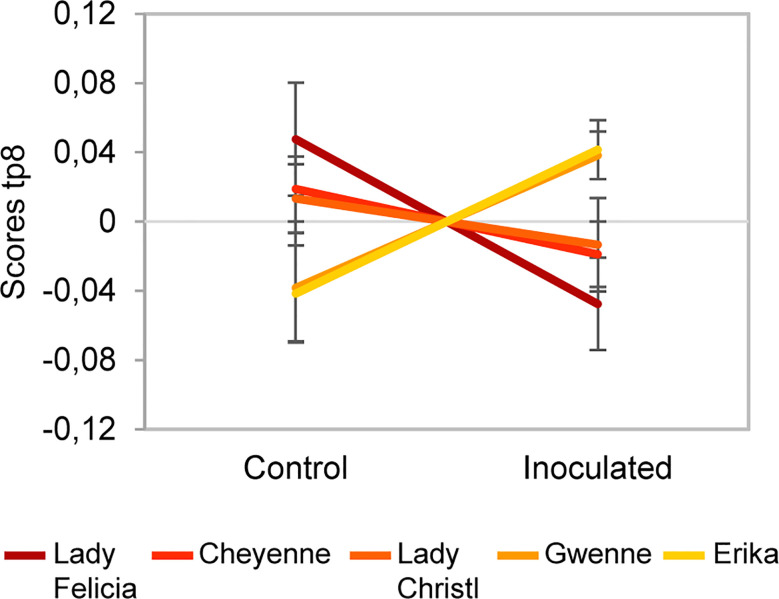
Scores of predictive component tp8 associated with the *Cultivar x Inoculation* interaction effect for the negative ion mode dataset. The score value is reported on the vertical axis for control and inoculated conditions (in the horizontal axis). A difference between resistant cultivars (Erika and Gwenne) and susceptible cultivars (Lady Felicia, Cheyenne, and Lady Christl) can be observed.

The AMOPLS models (NI and PI modes) showed that the interaction between cultivar and inoculation had a statistically significant effect on the metabolomic profile of potato tubers (**Supplementary Table S2**). The interaction effect was responsible for 6% of the total variability observed in this study (**Supplementary Table 2**). This result indicates that the cultivars respond differently to the inoculation of tubers with *C. coccodes* and suggest that resistance-related induced (RRI) biomarkers could be identified. The 8^th^ predictive component of the AMOPLS model indicates that some metabolites are differentially induced in the resistant versus the susceptible cultivars (**Figure 9**). It has been previously suggested that the response to pathogen attack in plants from the *Solanum* genera do not differ between compatible and incompatible interactions (Desender et al., 2007). However, our results suggest that potato cultivars with different susceptibility levels to black dot have different metabolic responses Nonetheless, this component highlights trends upon inoculation but does not take into account the constitutive amount of these metabolites. Quantitative resistance to bacterial and oomycete pathogens in potatoes has been defined by the final content of the target molecules rather than their inducibility by the pathogen (Kröner et al., 2012). Thus, two Resistance-Related Induced ratios were calculated: the relative induction ratio (qualitative RRI = (RP/RM)/(SP/SM)) and the relative concentration in inoculated tubers between resistant and susceptible cultivars (quantitative RRI=RP/SP) (**Supplementary Table 3**). Some clusters of the molecular network in PI mode highlighted steroid derivatives as quantitative RRIs (**Supplementary Figure S4**), with some features with positive fold changes in resistant cultivars and negative fold changes in susceptible cultivars upon inoculation (**Supplementary Table S3**). Interestingly, several hydroxycoumarins, which had been highlighted as induced metabolites upon *C. coccodes* inoculation, were found to be induced at higher levels in the resistant cultivars (**Supplementary Table S3**). Scopoletin has been shown to correlate with plant resistance against several pathogen in tobacco, and to possess antifungal activity (El Oirdi et al., 2010; Sun et al., 2014; Yang et al., 2018). Furthermore, several hydroxycoumarins have been associated with resistance to late blight in potato leaves and tubers (Yogendra et al., 2014; Yogendra et al., 2015; Hamzehzarghani et al., 2016). In our antifungal bioassay, scopoletin, and especially esculetin (which showed both high RRC and RRI scores), exhibited antifungal activity against *C. coccodes* at 500 µM (**Figure 8**). These results suggest that hydroxycoumarins are specifically induced upon fungal inoculation in black dot resistant cultivars and may limit the proliferation of the pathogen *in planta*.

## Conclusion

Quantitative resistance to plant pathogens is the result of several mechanisms that limit the progress of the pathogenic infection. Since it involves different processes, breaking this resistance is more complicated for the pathogen than in qualitative resistance, where a single mutation may be sufficient to overcome resistance. However, studying quantitative resistance involves complementary approaches to the traditional genetic screening for *R* genes. In this work, we studied both structural and biochemical mechanisms of potato cultivars with different degrees of quantitative resistance to black dot disease, and we found that both mechanisms seem to be involved at different degrees in quantitative resistance. From a structural point of view, we could clearly highlight across the selected cultivars that in general, phellem thickness does not correlate with black dot resistance. However, the potato cultivar with the thinnest skin, Lady Felicia, was the most susceptible suggesting that a minimum skin thickness may be required for quantitative resistance. This hypothesis is in accordance with previous studies in American cultivars but should be verified across a larger number of European cultivars. In addition, the skin suberin amounts or composition did not correlate with black dot resistance contrarily to that observed with other potato pathogens, such as *Streptomyces scabies*.

On the other hand, the metabolite composition of the five cultivars was found to differ significantly, and possible biomarkers of black dot resistance in potato tubers were identified as Resistant-Related Constitutive (RRC) metabolites. Among them, the glycoalkaloid alpha-chaconine, which had been previously shown to possess antifungal activities *in vitro* and to confer resistance to different potato diseases, seems to be involved in resistance to black dot. Other highlighted RRC were the HCAAs, which were previously found to be involved in cell wall fortification. In addition, we found that some HCAAs possess antifungal activities against *C. coccodes in vitro*, indicating a larger role of these metabolites in plant-pathogen interactions. Moreover, inoculation of *C. coccodes* induced the accumulation of RRI metabolites, particularly antifungal hydroxycoumarins, which were more prominent in all resistant cultivars. Altogether, our results suggest that metabolite composition is the main determinant of resistance of potato cultivars to black dot. The approach used could be applied to a wider panel of potato cultivars in order to confirm the trends observed in the cultivars investigated in this study. Since most of the highlighted biomarkers were found to be constitutively more abundant in the resistant cultivars, their role in resistance to other fungal diseases may be worth investigating. Potentially, these compounds could be used for Marker Assisted Selection within breeding programs and contribute to a sustainable production of table potatoes.

## Data Availability Statement

The datasets presented in this study can be found in online repositories. The names of the repository/repositories and accession number(s) can be found below: https://osf.io/x4dk9/, X4DK9 ftp://massive.ucsd.edu/MSV000085467/, MSV000085467.

## Author Contributions

JM-C, KG, StS, and J-LW conceived and designed the study. JM-C performed the experiments in the greenhouse and the *in vitro* antifungal assays. JM-C and SyS extracted the suberin and specialized metabolites and prepared them for analysis. EM conducted the microscopy analysis of the tuber periderm. MC performed the GC-MS analysis of suberin monomers and analyzed the data. P-MA performed the LCMS analysis of specialized metabolites. JB performed the multivariate data analysis (AMOPLS). JM-C, P-MA, and AR analyzed the LCMS data and performed the molecular network analysis. JM-C, SyS, JB, P-MA, AR, KG, and JL-W analyzed and interpreted the results. JM-C, KG, and JL-W wrote the manuscript. All authors contributed to the article and approved the submitted version.

## Funding

This work was supported by the Commission for Technology and Innovation (Kommission für Technologie und Innovation), Switzerland. Grant number 18536.1 PFLS-LS, Project title: Integrierte Bekämpfung von *Colletotrichum coccodes* und *Helminthosporium solani* in der Kartoffelwirtschaft.

## Conflict of Interest

The authors declare that the research was conducted in the absence of any commercial or financial relationships that could be construed as a potential conflict of interest.
