# Supplementary material for: Insights on the Structural and Metabolic Resistance of Potato (Solanum tuberosum) Cultivars to Tuber Black Dot (Colletotrichum coccodes)
Source: Front Plant Sci. 2020 Aug 20;11:1287. doi: 10.3389/fpls.2020.01287 (PMC7468465; doi:10.3389/fpls.2020.01287)
Supplement: Supplementary file 1 [file Image_1.pdf]

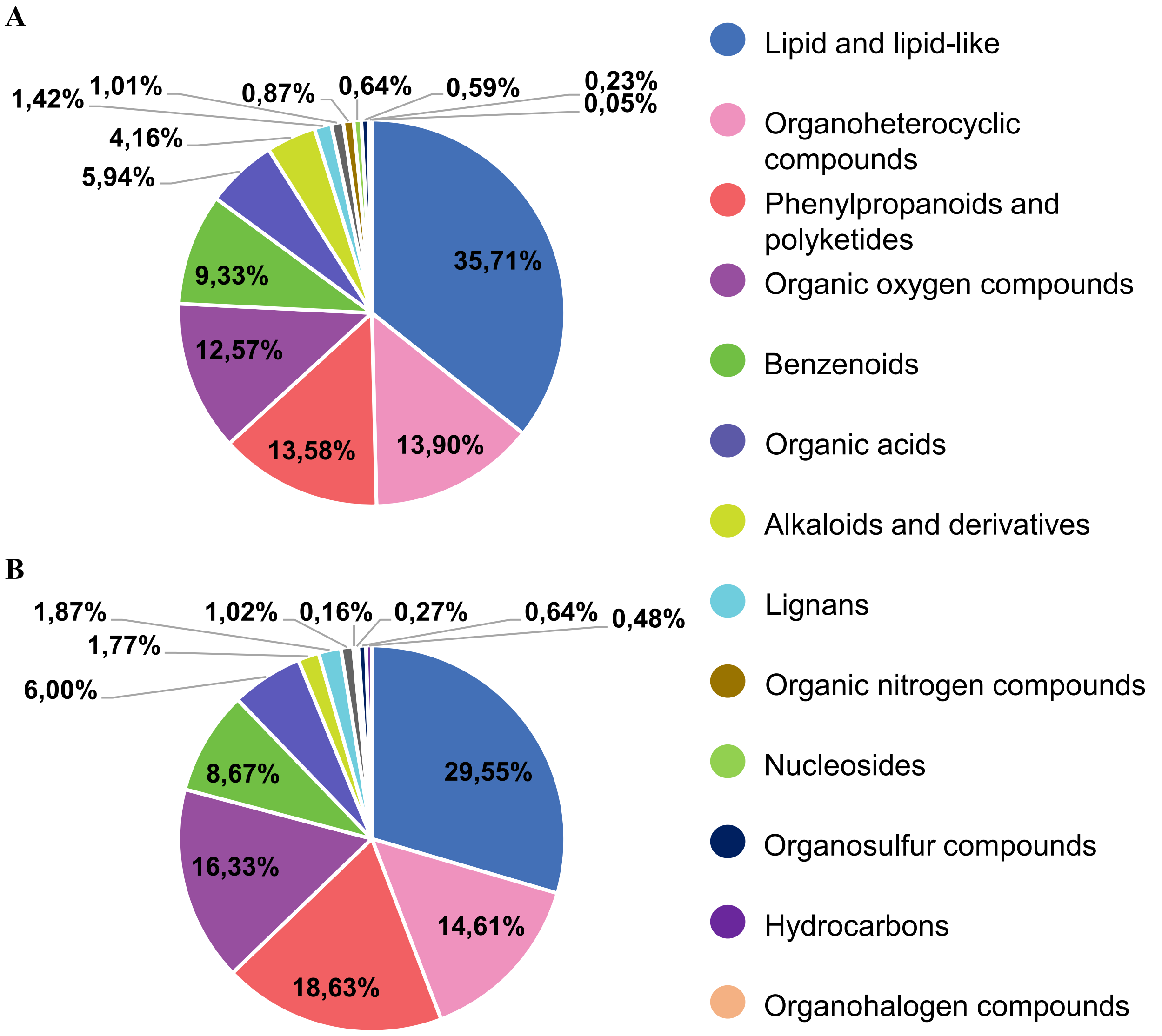

Supplementary Figure 1: Pie-chart of the chemical classes of compounds detected in potato periderms in positive (A) and negative (B) ionization modes as annotated by the ISDB-DNP and classified using ClassyFire.
