# Supplementary material for: Insights on the Structural and Metabolic Resistance of Potato (Solanum tuberosum) Cultivars to Tuber Black Dot (Colletotrichum coccodes)
Source: Front Plant Sci. 2020 Aug 20;11:1287. doi: 10.3389/fpls.2020.01287 (PMC7468465; doi:10.3389/fpls.2020.01287)
Supplement: Supplementary file 2 [file Image_2.pdf]

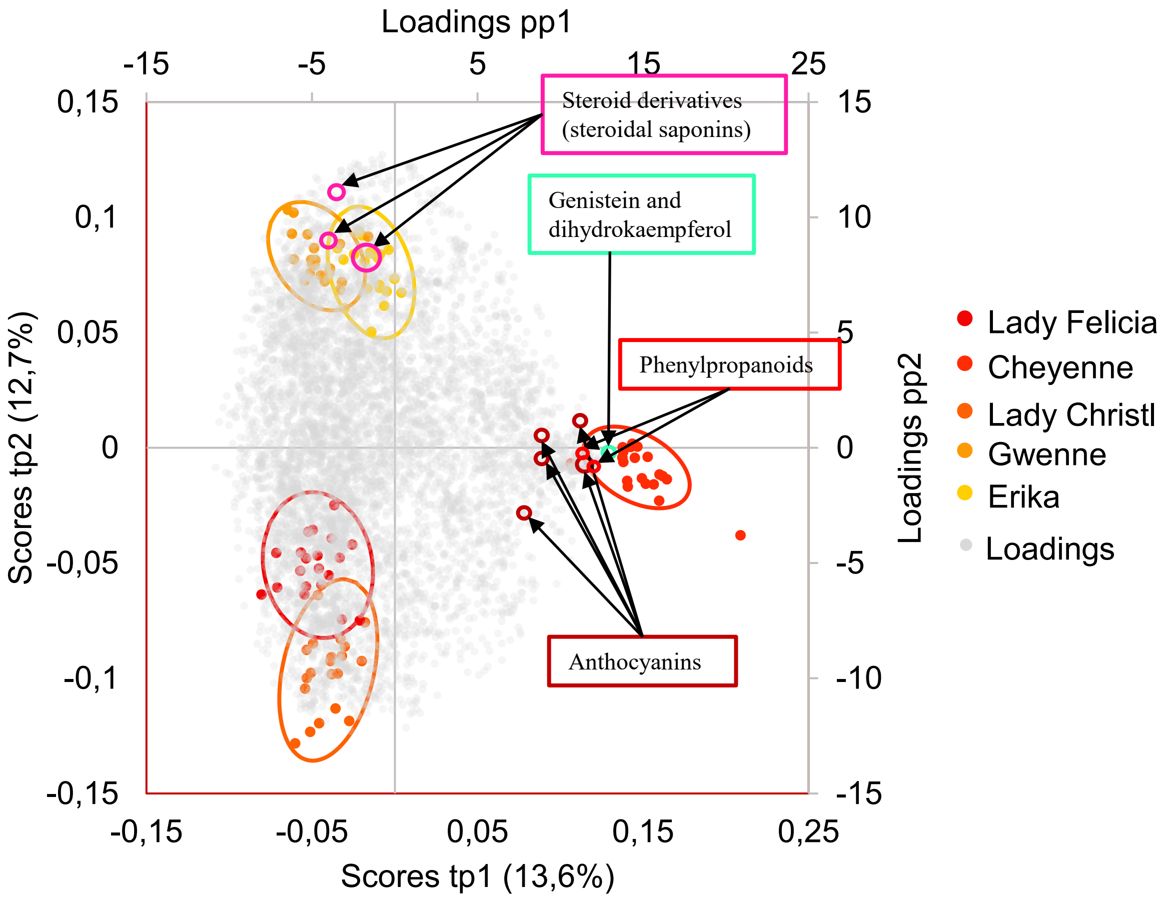

Supplementary Figure 2: Biplot representation of the two first predictive components of the AMOPLS model in positive ion mode explaining 26,4% of the total variance. Observations are positioned according to the left and below axis. Loadings are according to the right and above axis. Observations are color-coded as cultivars (n=20) with 95% confidence ellipses. The first component of the model (tp1) separates the red-skin cultivar (Cheyenne, positive scores) from the rest of the cultivars (negative scores). Resistant cultivars (Erika and Gwenne) have positive scores of tp2, while susceptible cultivars (Lady Felicia, Cheyenne and Lady Christl) have negative scores of tp2. The loadings of Cheyenne-specific metabolites annotated as anthocyanins, phenylpropanoids or the isoflavonoid genistein and the flavononol dihydrokaempferol are indicated. The loadings of highlighted resistance-related metabolites annotated as as steroid derivatives are also indicated.
