# Supplementary material for: Insights on the Structural and Metabolic Resistance of Potato (Solanum tuberosum) Cultivars to Tuber Black Dot (Colletotrichum coccodes)
Source: Front Plant Sci. 2020 Aug 20;11:1287. doi: 10.3389/fpls.2020.01287 (PMC7468465; doi:10.3389/fpls.2020.01287)
Supplement: Supplementary file 3 [file Image_3.pdf]

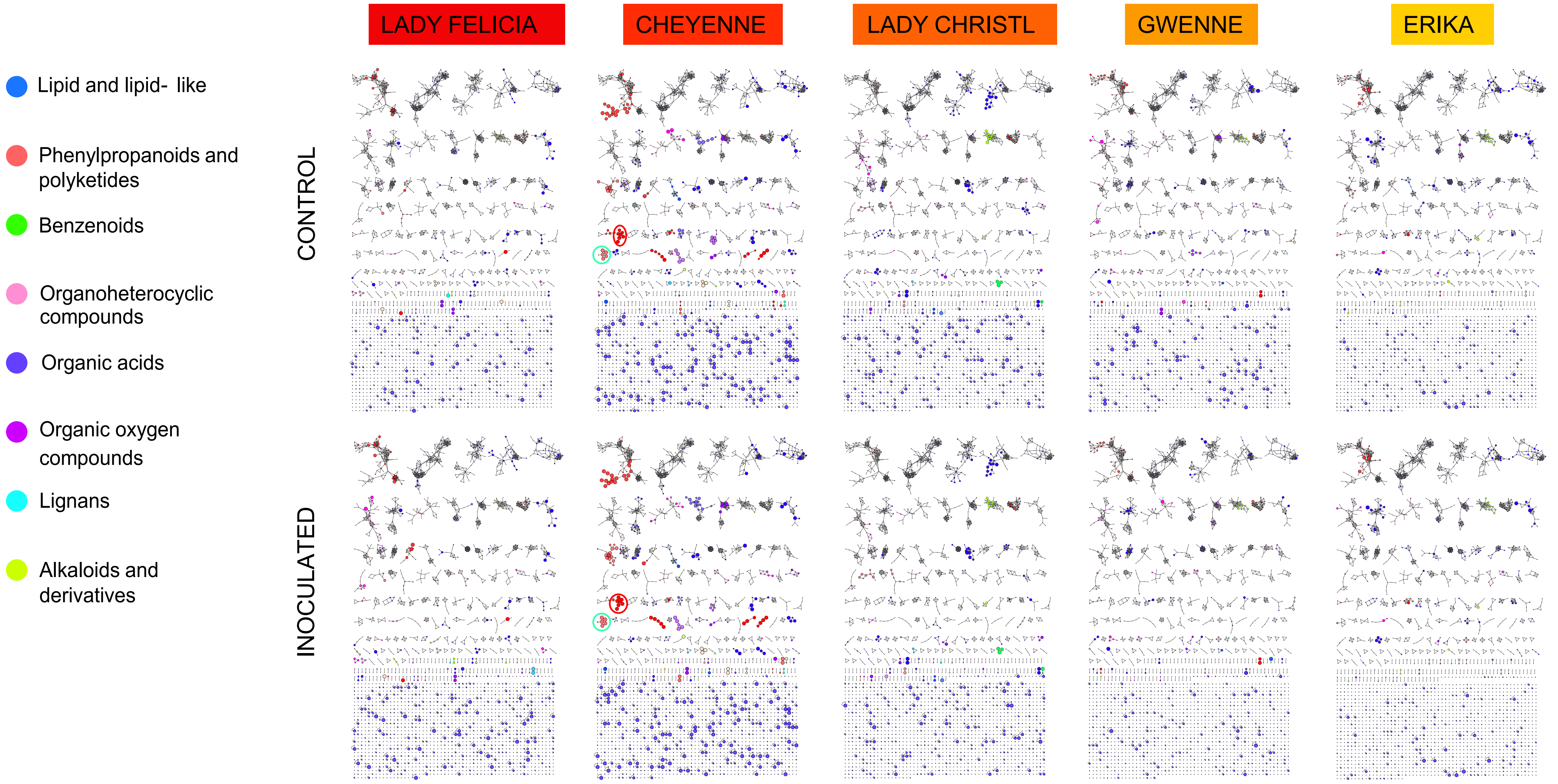

Supplementary Figure 3: Individual Molecular Networks in positive ion mode of the five potato cultivars in control and inoculated samples. Color of the node is set according to consensus chemical class across the cluster, transparency is set according to consistency of the chemical class annotation within the cluster, node size is set according to relative signal intensity in the respective cultivar. The red-skin cultivar (Cheyenne) appears to have the most abundant number of specific metabolites, including the isoflavonoid genistein and the flavononol dihydrokaempferol (in the cluster highlighted in light-blue), and a cluster of phenylpropanoids (the cluster highlighted in red) that was also highlighted in the AMOPLS model.
