# Supplementary material for: Insights on the Structural and Metabolic Resistance of Potato (Solanum tuberosum) Cultivars to Tuber Black Dot (Colletotrichum coccodes)
Source: Front Plant Sci. 2020 Aug 20;11:1287. doi: 10.3389/fpls.2020.01287 (PMC7468465; doi:10.3389/fpls.2020.01287)
Supplement: Supplementary file 4 [file Image_4.pdf]

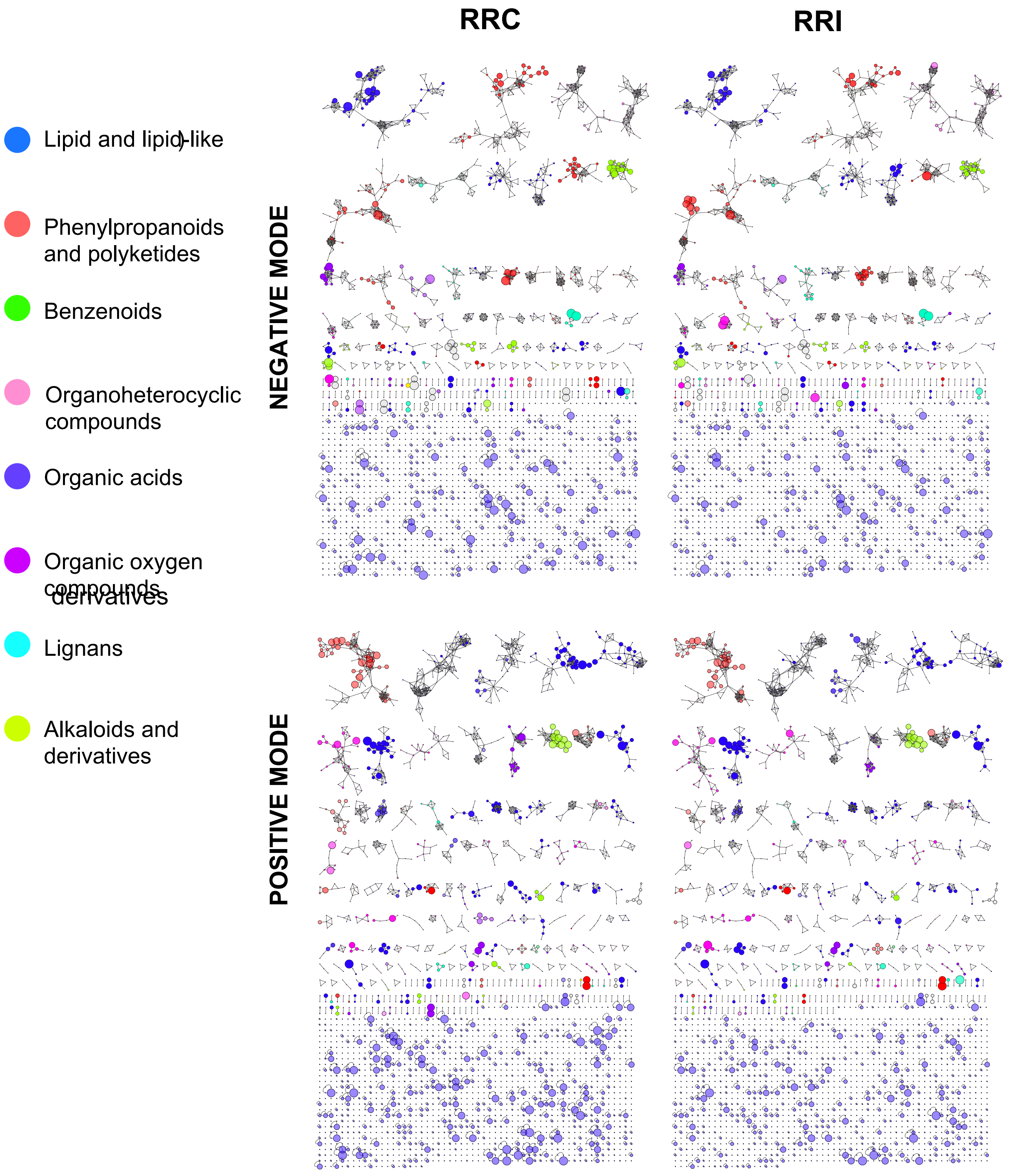

Supplementary Figure 4: Global Molecular Networks in positive and negative ion mode of the five potato cultivars. Color of the nose is set according to the consensus chemical across the cluster, transparency is set according to consistency of the chemical class annotation within the cluster, node size is set according to the ratio of intensities between resistnat and susceptible cultivars in control conditions (RRC) or in inoculated samples (RRI).
