# Supplementary material for: Insights on the Structural and Metabolic Resistance of Potato (Solanum tuberosum) Cultivars to Tuber Black Dot (Colletotrichum coccodes)
Source: Front Plant Sci. 2020 Aug 20;11:1287. doi: 10.3389/fpls.2020.01287 (PMC7468465; doi:10.3389/fpls.2020.01287)
Supplement: Supplementary file 5 [file Image_5.pdf]

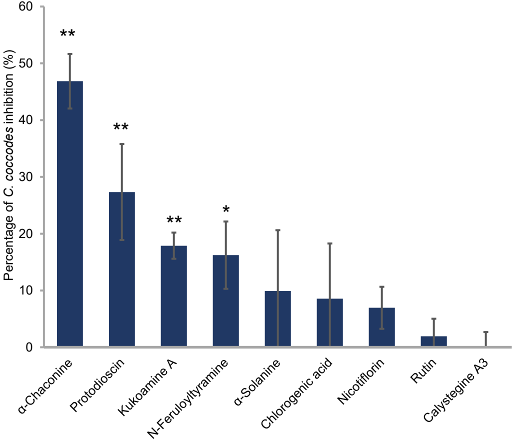

Supplementary Figure 5: Inhibition of *C. coccodes* growth (%) *in vitro* at the highest estimated concentration in potato tubers (n=3, Mean ± Standard deviation). Chlorogenic acid 7,5 mM, alpha-chaconine and calystegine A3 500 µM, alpha-solanine 250 µM, rutin and protodioscin 100 µM, N-feruloyltyramine, N1,N12-bis(dihydrocaffeoyl) spermine and nicotiflorin at 50 µM.
