# Supplementary material for: Insights on the Structural and Metabolic Resistance of Potato (Solanum tuberosum) Cultivars to Tuber Black Dot (Colletotrichum coccodes)
Source: Front Plant Sci. 2020 Aug 20;11:1287. doi: 10.3389/fpls.2020.01287 (PMC7468465; doi:10.3389/fpls.2020.01287)
Supplement: Supplementary file 6 [file Image_6.pdf]

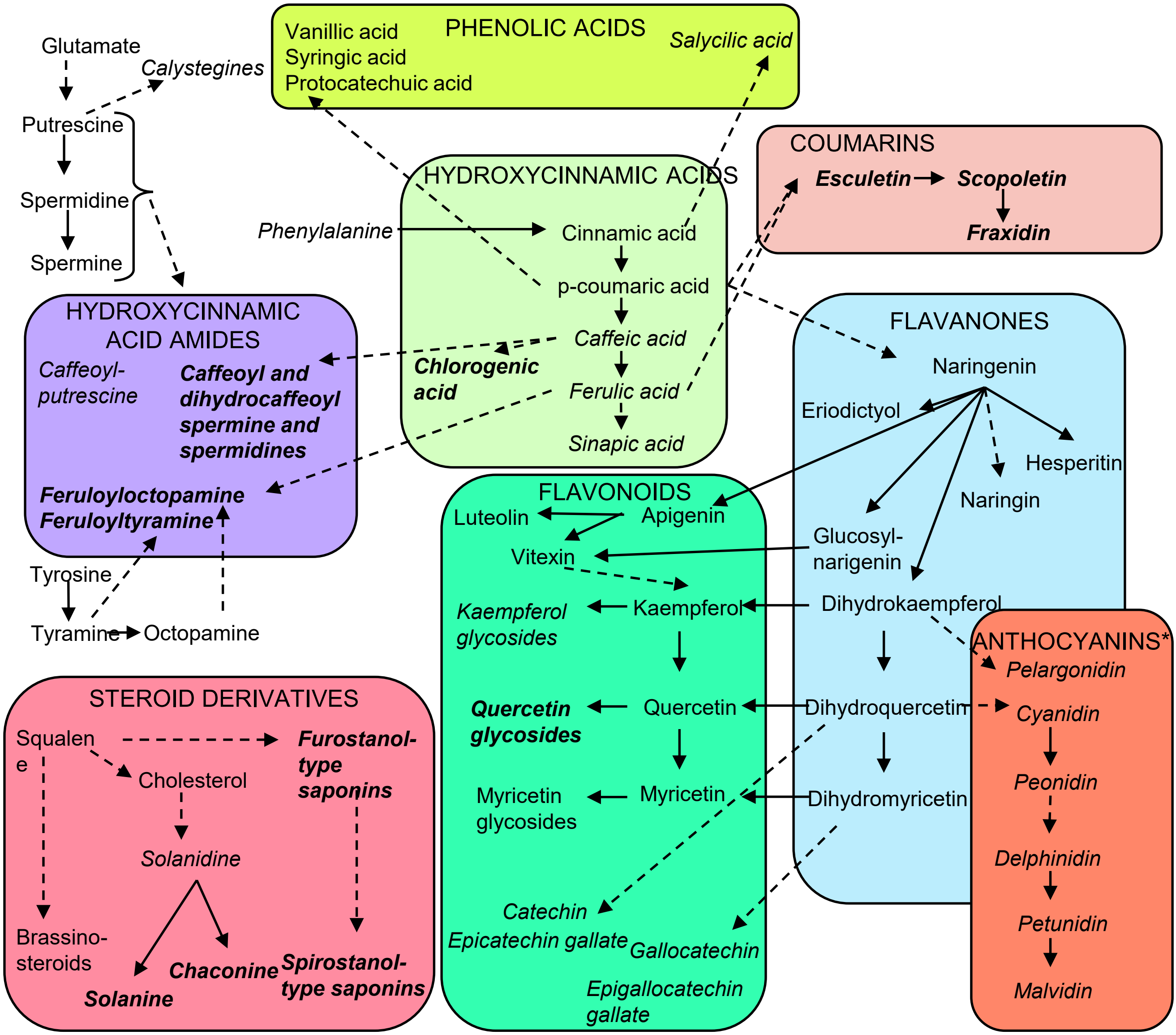

Supplementary Figure S6. Metabolic pathways involved in the interaction between *Solanum tuberosum* and *Colletotrichum coccodes*. The central cluster is formed by the hydroxycinnamic acids (HCAs) that derive from phenylalanine. Hydroxycinnamic acid amides (HCAAs) that derive from HCAs and amino acid derivatives form the cluster in the left. Cinnamic acid and *p*-coumaric acid are precursors of phenolic acids such as salicylic acid. Coumarins derive from *p*-coumaric acid or ferulic acid. Naringenin, produced from *p*-coumaric acid, is the precursor of flavonoids and anthocyanins. Steroidal glycoalkaloids (i.e. α-chaconine and α-solanine), steroidal saponins and brassinosteroids derive from squalene. *For anthocyanins, only the aglycone (anthocyanidin) is shown. Detected metabolites in this study are shown in italics. Dotted lines indicate that more than one step is required for the conversion shown in the figure. Biosynthetic pathways according to the Kyoto Encyclopedia of Genes and Genomes (KEGG) of *Solanum tuberosum* (or related plant species, if not available for *S. tuberosum*).
