# Supplementary material for: Insights on the Structural and Metabolic Resistance of Potato (Solanum tuberosum) Cultivars to Tuber Black Dot (Colletotrichum coccodes)
Source: Front Plant Sci. 2020 Aug 20;11:1287. doi: 10.3389/fpls.2020.01287 (PMC7468465; doi:10.3389/fpls.2020.01287)
Supplement: Supplementary file 7 [file Image_7.pdf]

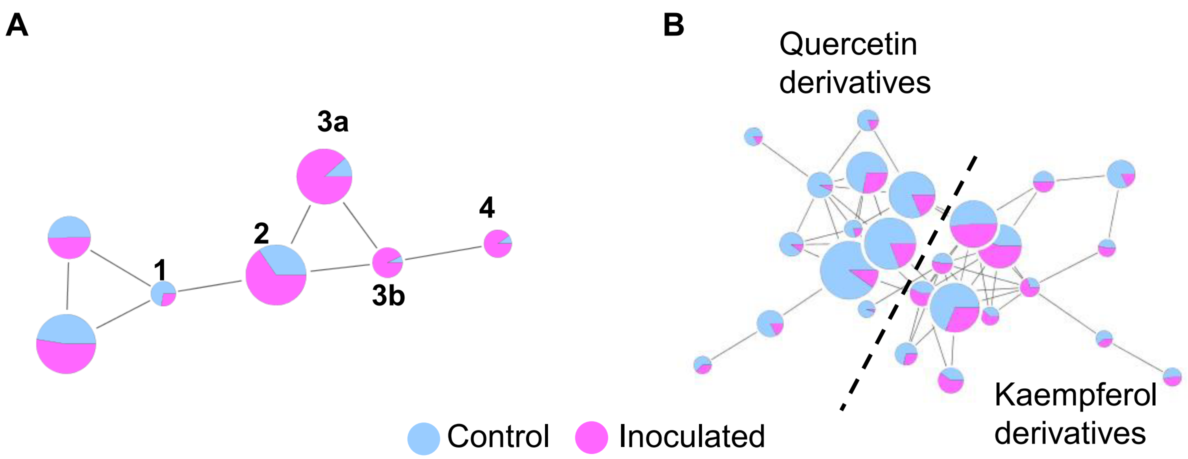

Supplementary Figure 7: Molecular Network clusters of hydroxycoumarins (A) and flavonoid glycosides (B); size of the node corresponds to mean peak intensity. The color of each node is set as a pie-chart with the relative abundance of the metabolite in control (light blue) or inoculated (pink) conditions In A: 1. Esculetin, 2. Scopoletin, 3a and 3b. Isofraxidin, 4. Dimethylfraxidin. In B: dotted line separates quercetin glycosides (left) and kaempferol glycosides (right).
