# Supplementary material for: Insights on the Structural and Metabolic Resistance of Potato (Solanum tuberosum) Cultivars to Tuber Black Dot (Colletotrichum coccodes)
Source: Front Plant Sci. 2020 Aug 20;11:1287. doi: 10.3389/fpls.2020.01287 (PMC7468465; doi:10.3389/fpls.2020.01287)
Supplement: Supplementary file 8 [file Table_1.docx]

**Supplementary Table 1**. Suberin monomeric composition in tuber periderm (µg/mg periderm) of the five potato cultivars studied (n=6, mean ± Standard deviation). ^a^ In brackets, percentage of each monomers class.

| **Compound** | Periderm (µg/mg) | | | | |
| --- | --- | --- | --- | --- | --- |
| *Aromatics* | **Lady Felicia** | **Cheyenne** | **Lady Christl** | **Gwenne** | **Erika** |
| Citric acid | 0,40 ± 0,06 | 0,44 ± 0,22 | 0,42 ± 0,23 | 0,71 ± 0,24 | 0,83 ± 0,34 |
| Vanillic acid | 0,76 ± 0,26 | 0,65 ± 0,08 | 0,43 ± 0,26 | 0,48 ± 0,09 | 0,76 ± 0,18 |
| p-coumaric acid | 0,14 ± 0,11 | 1,06 ± 0,54 | 0,11 ± 0,05 | 0,15 ± 0,07 | 0,16 ± 0,03 |
| Ferulic acid | 9,84 ± 1,18 | 9,89 ± 2,17 | 7,29 ± 1,6 | 8,25 ± 0,51 | 10,73 ± 2,04 |
| Caffeic acide | 1,26 ± 0,68 | 1,57 ± 0,83 | 1,17 ± 1,07 | 0,87 ± 0,51 | 1,61 ± 0,42 |
| **TOTAL Aromatics^a^** | **12,39 ± 1,75 (12,1%)** | **13,61 ± 3,32 (14,8%)** | **9,42 ± 2,94 (10,1%)** | **10,47 ± 0,62 (9,8%)** | **14,10 ± 2,3 (12,6%)** |
| *Alkanoic acids* |  |  |  |  |  |
| Hexadecanoic acid (C16:0) | 0,44 ± 0,06 | 0,35 ± 0,05 | 0,29 ± 0,06 | 0,38 ± 0,05 | 0,53 ± 0,07 |
| Octadecanoic acid (C18:0) | 0,08 ± 0,02 | 0,07 ± 0,04 | 0,08 ± 0,01 | 0,06 ± 0,01 | 0,06 ± 0,01 |
| Docosanoic acid (C22:0) | 0,14 ± 0,09 | 0,28 ± 0,06 | 0,31 ± 0,04 | 0,36 ± 0,06 | 0,19 ± 0,02 |
| Tetracosanoic acid (C24:0) | 1,18 ± 0,34 | 1,05 ± 0,29 | 1,49 ± 0,2 | 1,66 ± 0,21 | 1,58 ± 0,31 |
| Pentacosanoic acid (C25:0) | 0,52 ± 0,11 | 0,48 ± 0,28 | 0,59 ± 0,05 | 0,64 ± 0,07 | 0,33 ± 0,07 |
| Hexacosanoic acid (C26:0) | 3,32 ± 0,31 | 2,81 ± 0,7 | 3,45 ± 0,34 | 3,15 ± 0,3 | 3,35 ± 0,41 |
| Heptacosanoic acid (C27:0) | 0,21 ± 0,14 | 0,18 ± 0,06 | 0,26 ± 0,03 | 0,08 ± 0,02 | 0,14 ± 0,04 |
| Octacosanoic acid (C28:0) | 16,09 ± 1,97 | 9,78 ± 2,15 | 10,40 ± 1,62 | 7,10 ± 0,76 | 9,95 ± 0,92 |
| Nonacosanoaic acid (C29:0) | 4,09 ± 0,48 | 2,75 ± 0,6 | 2,52 ± 0,43 | 1,47 ± 0,23 | 2,56 ± 0,59 |
| Triacontanoic acid (C30:0) | 11,77 ± 1,99 | 7,94 ± 1,68 | 5,98 ± 0,96 | 12,50 ± 1,94 | 10,87 ± 0,75 |
| **TOTAL Alkanoic acids^a^** | **37,83 ± 5,17 (36,9%)** | **25,68 ± 5,35 (28,0%)** | **25,36 ± 3,25 (27,3%)** | **27,41 ± 3,56 (25,7%)** | **29,55 ± 2,36 (26,4%)** |
| *1-alkanols* |  |  |  |  |  |
| Octadecanol | 0,17 ± 0,05 | 0,22 ± 0,1 | 0,17 ± 0,08 | 0,15 ± 0,03 | 0,11 ± 0,11 |
| Eicosanol | 0,04 ± 0,01 | 0,10 ± 0,03 | 0,08 ± 0,04 | 0,06 ± 0,01 | 0,11 ± 0,04 |
| Heneicosanol | 0,67 ± 0,23 | 0,90 ± 0,25 | 0,66 ± 0,07 | 0,81 ± 0,05 | 0,70 ± 0,17 |
| Docosanol | 0,42 ± 0,11 | 0,69 ± 0,22 | 0,48 ± 0,2 | 0,53 ± 0,03 | 0,25 ± 0,09 |
| Tetracosanol | 0,57 ± 0,1 | 0,55 ± 0,14 | 0,60 ± 0,19 | 0,84 ± 0,05 | 0,68 ± 0,39 |
| Hexacosanol | 1,48 ± 0,54 | 1,26 ± 0,28 | 1,64 ± 0,4 | 1,79 ± 0,12 | 2,02 ± 0,25 |
| Octacosanol | 4,75 ± 1,92 | 3,89 ± 1,02 | 4,05 ± 0,91 | 4,39 ± 0,37 | 4,36 ± 0,88 |
| Tricontanol | 0,38 ± 0,14 | 0,51 ± 0,11 | 0,41 ± 0,24 | 0,75 ± 0,11 | 0,59 ± 0,09 |
| **TOTAL 1-alkanols^a^** | **8,48 ± 2,81 (8,3%)** | **8,11 ± 1,83 (8,8%)** | **8,09 ± 1,49 8,7%)** | **9,31 ± 0,66 (7,7%)** | **8,80 ± 1,25 (7,8%)** |
| α,ω-Diacids |  |  |  |  |  |
| Hexadecanedioic acid | 0,93 ± 0,26 | 1,42 ± 0,42 | 1,02 ± 0,16 | 1,27 ± 0,13 | 1,07 ± 0,24 |
| Octadecanedioic acid | 1,43 ± 0,42 | 1,34 ± 0,42 | 1,81 ± 0,27 | 2,10 ± 0,15 | 1,55 ± 0,35 |
| Octadec-9-enedioic acid | 15,02 ± 3,99 | 15,07 ± 1,68 | 17,99 ± 3,91 | 22,14 ± 1,87 | 19,54 ± 4,27 |
| Eicosanedioic acid | 0,42 ± 0,18 | 0,33 ± 0,14 | 0,54 ± 0,14 | 0,57 ± 0,03 | 0,42 ± 0,11 |
| Docosanedioic acid | 0,50 ± 0,28 | 0,79 ± 0,45 | 0,87 ± 0,34 | 0,96 ± 0,07 | 0,70 ± 0,32 |
| Tetracosanedioic acid | 1,01 ± 0,25 | 1,17 ± 0,43 | 1,46 ± 0,41 | 1,64 ± 0,27 | 1,55 ± 0,26 |
| Hexacosanedioic acid | 0,71 ± 0,14 | 0,61 ± 0,28 | 1,04 ± 0,34 | 1,20 ± 0,15 | 1,22 ± 0,24 |
| **Total α,ω-Diacids^a^** | **20,04 ± 4,59 (19,6%)** | **20,73 ± 3,19 (22,6%)** | **24,72 ± 4,46 (26,6%)** | **29,86 ± 1,94 (27,9%)** | **26,05 ± 4,56 (23,2%)** |
| *Hydroxyacids* |  |  |  |  |  |
| 2-Hydroxyhexadecanoic acid | 0,11 ± 0,05 | 0,11 ± 0,04 | 0,14 ± 0,05 | 0,14 ± 0,02 | 0,17 ± 0,05 |
| 16-Hydroxyhexadecanoic acid | 0,60 ± 0,2 | 0,91 ± 0,28 | 0,71 ± 0,15 | 1,08 ± 0,17 | 0,92 ± 0,22 |
| 18-Hydroxyoctadecanoic acid | 0,02 ± 0,01 | 0,03 ± 0,01 | 0,03 ± 0,01 | 0,03 ± 0,01 | 0,03 ± 0,01 |
| 18-Hydroxy-octadec-9-enoate | 16,68 ± 5,96 | 16,04 ± 4,74 | 16,19 ± 2,75 | 18,27 ± 1,31 | 23,21 ± 7,29 |
| 20-Hydroxyeicosanoic acid | 0,14 ± 0,24 | 0,03 ± 0,02 | 0,06 ± 0,05 | 0,03 ± 0,02 | 0,12 ± 0,13 |
| 22-Hydroxydocosanoic acid | 0,82 ± 0,25 | 1,43 ± 0,4 | 1,02 ± 0,44 | 1,39 ± 0,2 | 0,75 ± 0,47 |
| 24-Hydroxytetracosanoic acid | 2,33 ± 1,14 | 1,83 ± 0,97 | 3,01 ± 0,89 | 4,15 ± 0,72 | 3,16 ± 1,19 |
| 26-Hydroxyhexacosanoic acid | 2,04 ± 0,81 | 1,85 ± 0,49 | 2,83 ± 0,62 | 3,08 ± 0,27 | 2,87 ± 0,51 |
| 28-Hydroxyoctacosanoic acid | 1,02 ± 0,48 | 1,39 ± 0,44 | 1,43 ± 0,44 | 1,65 ± 0,17 | 1,29 ± 0,6 |
| **Total Hydroxyacids^a^** | **23,76 ± 7,86 (23,2%)** | **23,61 ± 5,15 (25,7%)** | **25,43 ± 5,02 (27,3%)** | **29,82 ± 1,89 (27,9%)** | **33,61 ± 8,81 (30,0%)** |
| **TOTAL SUBERIN** | **102,50 ± 22,72** | **91,74 ± 19,38** | **93,03 ± 17,43** | **106,86 ± 9,48** | **112,11 ± 19,99** |
