# Supplementary material for: Insights on the Structural and Metabolic Resistance of Potato (Solanum tuberosum) Cultivars to Tuber Black Dot (Colletotrichum coccodes)
Source: Front Plant Sci. 2020 Aug 20;11:1287. doi: 10.3389/fpls.2020.01287 (PMC7468465; doi:10.3389/fpls.2020.01287)
Supplement: Supplementary file 9 [file Table_2.docx]

**Supplementary Table 2**. ANOVA decomposition and AMOPLS predictive components (*tp1* to *to*, first row) related to the specific effects of the experimental design (*Cultivar, Inoculation or Cultivar × Inoculation*), for the LC-HRMS/MS dataset (Positive and Negative Ionization, respectively). The predictive components associated with each specific effect are highlighted.

POSITIVE MODE

| **ANOVA decomposition** | **Cultivar** | | | | **Inno-culation** | **Interaction** | | | | **Residuals** |
| --- | --- | --- | --- | --- | --- | --- | --- | --- | --- | --- |
| **Relative Sum of Squares** | 48,3% | | | | 4,8% | 5,5% | | | | 41,5% |
| Predictive components | tp1 | tp2 | tp3 | tp4 | tp5 | tp6 | tp7 | tp8 | tp9 | to |
| *Cultivar* | **96,10%** | **96,90%** | **97,40%** | **91,50%** | 1,60% | 2,90% | 5,80% | 6,10% | 7,20% | 10,00% |
| *Inoculation* | 1,20% | 0,90% | 0,80% | 2,50% | **87,90%** | 7,90% | 15,80% | 16,40% | 19,60% | 27,00% |
| *Cultivar × Inoculation* | 1,30% | 1,00% | 0,90% | 2,80% | 4,90% | **79,50%** | **58,90%** | **57,20%** | **49,00%** | 29,60% |
| *Residuals* | 1,40% | 1,20% | 1,00% | 3,10% | 5,50% | 9,70% | 19,50% | 20,30% | 24,20% | 33,40% |

NEGATIVE MODE

| **ANOVA decomposition** | **Cultivar** | | | | **Inno-culation** | **Interaction** | | | | **Residuals** |
| --- | --- | --- | --- | --- | --- | --- | --- | --- | --- | --- |
| **Relative Sum of Squares** | 47,8% | | | | 4,5% | 5,6% | | | | 42,1% |
| Predictive components | tp1 | tp2 | tp3 | tp4 | tp5 | tp6 | tp7 | tp8 | tp9 | to |
| *Cultivar* | **96,60%** | **97,90%** | **90,80%** | **96,20%** | 2,80% | 2,70% | 2,80% | 6,70% | 7,90% | 10,10% |
| *Inoculation* | 1,00% | 0,60% | 2,80% | 1,20% | **80,10%** | 7,40% | 7,70% | 18,20% | 21,50% | 27,30% |
| *Cultivar × Inoculation* | 1,10% | 0,70% | 3,00% | 1,20% | 8,00% | **80,80%** | **80,00%** | **52,90%** | **44,50%** | 29,30% |
| *Residuals* | 1,30% | 0,80% | 3,40% | 1,40% | 9,10% | 9,00% | 9,40% | 22,20% | 26,10% | 33,30% |
