# Supplementary material for: Insights on the Structural and Metabolic Resistance of Potato (Solanum tuberosum) Cultivars to Tuber Black Dot (Colletotrichum coccodes)
Source: Front Plant Sci. 2020 Aug 20;11:1287. doi: 10.3389/fpls.2020.01287 (PMC7468465; doi:10.3389/fpls.2020.01287)
Supplement: Supplementary file 10 [file Table_3.docx]

**Supplementary Table 3**. List of metabolites detected in the untargeted metabolomics approach and highlighted as resistance-related metabolites or cultivar-specific metabolites. NI Negative ionization mode; PI Positive Ionization mode. RT: Retention Time. Chemical class according to ClassyFyre (Subclass or parent). The values of each feature are standardized to the lowest intensity peak of the feature. RRC=RM/SM, RRI=RP/SP, qualitativeRRI =(RP/RM)/(SP/SM). RRC resistance-related constitutive, RRI resistance-related induced, RP resistant genotype with pathogen inoculation, RM resistant genotype with mock inoculation, SP susceptible genotype with pathogen inoculation, SM susceptible genotype with mock inoculation. Annotation: 1: pure standard (HRMS, MS/MS, RT); 2a: GNPS experimental data; 2b: in silico database (ISDB) with taxonomic ponderation; 3: comparison with published data (anthocyanins (Ieri et al. 2011), steroidal saponins and HCAAs (Parr et al. 2005; Huang et al. 2017); 4: Unannotated features from an annotated MN cluster; 5: HRMS (no MS/MS data available).

| m/z  ratio | Mode | RT | Putative  idendity | Chemical  Class | Lady Felicia | | Cheyenne | | Lady Christl | | Gwenne | | Erika | | RRC | RRI | qRRI | Anno- tation |
| --- | --- | --- | --- | --- | --- | --- | --- | --- | --- | --- | --- | --- | --- | --- | --- | --- | --- | --- |
|  |  |  |  |  | C | I | C | I | C | I | C | I | C | I |  |  |  |  |
| 164,0708 | NI | 0,54 | Phenylalanine | Aminoacid | 2,64 | 1,94 | 1,05 | 1,00 | 4,91 | 2,57 | 4,26 | 3,75 | 1,53 | 1,39 | **1,01** | **1,40** | **1,38** | **1** |
| 801,2946 | PI | 1,75 | mal 3-O-rut-5- O-glu | Antho- cyanins | 3,79 | 4,12 | 1,00 | 1,93 | 7,19 | 7,24 | 6,18 | 6,30 | 5,02 | 3,64 | **1,40** | **1,12** | **0,80** | **2b** |
| 963,2770 | PI | 1,38 | pet 3-O-ferul -rut-5-O-glu | Antho- cyanins | 6,46 | 1,00 | nd | nd | nd | nd | nd | nd | nd | nd | **0,00** | **0,00** |  | **2b** |
| 933,2650 | PI | 1,31 | pet 3-O-p-coum -rut-5-O-glu | Antho- cyanins | 220,67 | 13,06 | 33,88 | 106,60 | 5,27 | 1,00 | nd | nd | 49,64 | 14,56 | **0,29** | **0,18** | **0,63** | **3** |
| 741,2236 | PI | 0,70 | pel 3-O-rut -5-O-glu | Antho- cyanins | nd | nd | 18,81 | 35,50 | nd | nd | nd | nd | 1,29 | 1,00 | **0,10** | **0,04** | **0,41** | **2b** |
| 579,1690 | PI | 1,05 | pel 3-O-rut | Antho- cyanins | 7,50 | 5,69 | 6,49 | 29,14 | 5,05 | 4,90 | 1,00 | 1,48 | 4,05 | 3,31 | **0,40** | **0,18** | **0,45** | **3** |
| 903,2545 | PI | 1,27 | pel 3-O-caf- rut-5-O-glu | Antho- cyanins | 7,49 | nd | 85,49 | 316,97 | 3,91 | 1,00 | nd | nd | 18,74 | 2,52 | **0,29** | **0,01** | **0,04** | **3** |
| 887,2606 | PI | 1,39 | pel 3-O-cis- p-coum-rut-5-O-glu | Antho- cyanins | 1,05 | nd | 1153,57 | 3784,59 | 3,88 | 1,00 | nd | nd | 13,54 | 4,34 | **0,02** | **0,00** | **0,10** | **3** |
| 887,2605 | PI | 1,68 | pel 3-O-p- coum-rut-5-O-glu | Antho- cyanins | nd | nd | 1,00 | 2,05 | nd | nd | nd | nd | nd | nd | **0,00** | **0,00** |  | **3** |
| 917,2705 | PI | 1,44 | pel 3-O-ferul  rut-5-O-glu | Antho- cyanins | 137,06 | 1,00 | 4080,06 | ####### | 90,94 | 24,45 | 14,95 | 15,67 | 578,56 | 79,46 | **0,21** | **0,01** | **0,06** | **3** |
| 947,2810 | PI | 1,49 | peo 3-O-ferul  rut-5-O-glu | Antho- cyanins | 18,48 | 1,00 | 128,53 | 246,35 | 18,16 | 5,63 | 6,18 | 7,92 | 78,53 | 34,48 | **0,77** | **0,25** | **0,33** | **2b** |
| 725,2069 | PI | 1,55 | pel 3-O-p-coum -rut | Antho- cyanins | nd | nd | 1,00 | 3,86 | nd | nd | nd | nd | nd | nd | **0,00** | **0,00** |  | **2b** |
| 755,2168 | PI | 1,62 | pel 3-O-ferul -rut | Antho- cyanins | 19,81 | 1,00 | 90,24 | 296,23 | 6,23 | 2,12 | 1,37 | nd | 60,30 | 16,00 | **0,80** | **0,08** | **0,10** | **3** |
| 191,0187 | NI | 0,31 | Citric acid | Carboxylic  acid | 1,43 | 1,00 | 1,59 | 1,55 | 1,88 | 1,13 | 1,92 | 1,93 | 1,29 | 1,71 | **1,13** | **1,22** | **1,08** | **2a** |
| 133,0128 | NI | 0,26 | Malic acid | Carboxylic  acid | 1,52 | 1,35 | 1,36 | 1,73 | 1,24 | 1,00 | 1,59 | 1,40 | 1,10 | 1,17 | **0,98** | **0,94** | **0,96** | **2a** |
| 147,0286 | NI | 0,33 | Citramalic acid | Carboxylic  acid | 1,00 | 1,27 | 1,17 | 2,77 | 1,56 | 1,63 | 1,70 | 1,14 | 1,12 | 1,32 | **1,13** | **0,65** | **0,57** | **2a** |
| 299,0775 | NI | 0,84 | Salicylate  2-O-beta-D-glucoside | Carboxylic  acid | 1,81 | 2,98 | 11,05 | 3,89 | 1,53 | 1,00 | 2,30 | 1,62 | 1,11 | 1,29 | **0,35** | **0,56** | **1,57** | **2b** |
| 917,5132 | NI | 2,72 | (1α,3β,7β,8α,9β,24S)-1,3,7,24- Tetrahydroxy-9,19- cyclolanostan-25-yl 4-O-{4-O-[(2S,3R,4R)- 3,4-dihydroxy-4- (hydroxymethyl)tetrahydro -2-furanyl]-β-D-xylopyranosyl} -β-D-glucopyranoside | Cyclo- artanols  and  derivatives | 75,78 | 47,76 | 1,03 | 1,25 | 1,00 | nd | 5,35 | 3,21 | 124,08 | 82,15 | **2,50** | **2,61** | **1,05** | **2b** |
| 447,0932 | NI | 1,53 | kaempferol 3-O-beta-D- galactoside | Flavonoid glycoside | 4,08 | nd | 726,14 | 1102,10 | nd | nd | 1,25 | 1,00 | 42,06 | 26,65 | **0,09** | **0,04** | **0,42** | **2b** |
| 463,0888 | NI | 1,41 | quercetin-3-O-glucoside  (isoquercitrin) | Flavonoid glycoside | 19,90 | nd | 1,43 | 1,00 | 8,87 | 2,67 | 13,68 | 3,88 | 65,35 | 6,20 | **3,92** | **4,12** | **1,05** | **2a/2b** |
| 591,1351 | NI | 1,69 | Kaempferol  derivative | Flavonoid glycoside | nd | nd | 21,91 | 12,70 | nd | nd | 1,00 | 1,46 | nd | nd | **0,07** | **0,17** | **2,53** | **4** |
| 593,1511 | NI | 1,51 | Kaempferol  3-O-Rutinoside | Flavonoid glycoside | 55,96 | 1,00 | 120,06 | 130,17 | 12,14 | 4,33 | 10,33 | 5,00 | 184,37 | 19,64 | **1,55** | **0,27** | **0,18** | **1** |
| 609,1469 | NI | 1,31 | Quercetin  3-O-Rutinoside | Flavonoid glycoside | 135,29 | 1,00 | 43,30 | 50,18 | 3,10 | 1,22 | 3,67 | 1,82 | 167,67 | 5,87 | **1,41** | **0,22** | **0,16** | **1** |
| 625,1418 | NI | 1,16 | Quercetin  Disaccharide | Flavonoid glycoside | 1,00 | nd | 20,78 | 17,77 | 14,98 | 6,80 | 30,73 | 8,75 | 27,89 | 2,53 | **2,39** | **0,69** | **0,29** | **2b** |
| 739,1891 | NI | 2,08 | Kaempferol-3-O-D- (2-O-trans-coumaroyl)-rutinoside | Flavonoid glycoside | 27,26 | nd | 429,88 | 780,95 | 11,50 | 4,47 | 5,74 | 1,00 | 147,61 | 15,63 | **0,49** | **0,03** | **0,06** | **4** |
| 753,1888 | NI | 1,43 | Kaempferol  derivative | Flavonoid glycoside | nd | nd | 119,62 | 101,86 | nd | nd | 1,00 | 3,81 | nd | nd | **0,01** | **0,06** | **4,47** | **4** |
| 755,1836 | NI | 1,94 | Quercetin 3-O-D- (2-O-trans-coumaroyl)-rutinoside | Flavonoid glycoside | 40,98 | nd | 24,40 | 40,95 | nd | nd | 1,00 | nd | 140,76 | 5,45 | **3,25** | **0,20** | **0,06** | **2b** |
| 755,2052 | NI | 1,21 | Kaempferol 3-O-rutinoside- 7-glucoside | Flavonoid glycoside | nd | nd | 119,21 | 129,39 | 6,48 | 2,63 | 10,72 | 3,07 | 8,92 | 1,00 | **0,23** | **0,05** | **0,20** | **4** |
| 771,1999 | NI | 1,10 | Quercetin 3-O-Rutinoside- 7-glucoside | Flavonoid glycoside | 1,00 | nd | 53,54 | 27,24 | 21,00 | 10,06 | 84,85 | 13,80 | 90,42 | 2,55 | **3,48** | **0,66** | **0,19** | **2a** |
| 785,1934 | NI | 2,03 | Quercetin  derivative | Flavonoid glycoside | 11,31 | nd | 1,00 | nd | nd | nd | 1,97 | 1,18 | 36,62 | 1,33 | **4,70** | **∞** | **∞** | **4** |
| 901,2429 | NI | 1,87 | Kaempferol-3-O-D- (2-O-trans-coumaroyl)- rutinoside-7-glucoside | Flavonoid glycoside | nd | nd | 1,00 | 2,39 | nd | nd | nd | nd | nd | nd | **0,00** | **0,00** | **0,00** | **4** |
| 931,2531 | NI | 1,94 | Kaempferol  triglycosides | Flavonoid glycoside | nd | nd | 153,92 | 238,69 | 1,00 | nd | nd | nd | nd | nd | **0,00** | **0,00** | **0,00** | **4** |
| 477,1042 | NI | 1,62 | (1S)-1,5-Anhydro-1- [3,5,7-trihydroxy-2- (4-hydroxyphenyl)-4-oxo-4H- chromen-8-yl]-D-threo-hexitol | Flavonoid glycoside | 1,49 | 0,00 | 7,99 | 8,26 | 1,00 | 0,00 | 1,60 | 1,61 | 16,07 | 7,21 | **2,53** | **1,60** | **0,63** | **2b** |
| 623,1619 | NI | 1,56 | isorhamnetin-3-O-rutinoside | Flavonoid glycoside | 23,57 | 1,00 | 4,88 | 3,65 | 3,54 | 2,52 | 5,83 | 2,78 | 23,38 | 2,68 | **1,37** | **1,14** | **0,83** | **2a/ 2b** |
| 655,1515 | NI | 1,29 | 3-[4,5-dihydroxy-6- (hydroxymethyl)-3- [3,4,5-trihydroxy-6- (hydroxymethyl)oxan-2-yl] oxyoxan-2-yl]oxy-5,7-dihydroxy- 2-(4-hydroxyphenyl)chromen- 4-one | Flavonoid glycoside | 0,00 | 0,00 | 127,02 | 153,36 | 2,54 | 1,00 | 3,79 | 0,00 | 0,00 | 0,00 | **0,04** | **0,00** | **0,00** | **2a/ 2b** |
| 769,2001 | NI | 2,14 | 4',5,7-Trihydroxy-3'- methoxyflavone | Flavonoid glycoside | 1,00 | 0,00 | 41,46 | 55,38 | 0,00 | 0,00 | 0,00 | 0,00 | 5,95 | 0,00 | **0,21** | **0,00** | **0,00** | **2b** |
| 839,1873 | NI | 1,09 | Quercetin 3-glycosides  Trisaccharides, 3-O-[β-D-Glucopyranosyl-(1→2)-[α-L-rhamnopyranosyl-(1→6)]-β-D-glucopyranoside] | Flavonoid glycoside | 0,00 | 0,00 | 25,96 | 13,70 | 10,82 | 4,65 | 39,93 | 7,12 | 36,66 | 1,00 | **3,12** | **0,66** | **0,21** | **2b** |
| 677,1333 | NI | 1,36 | 3',4,4',5',6-Pentahydroxyaurone;  (E)-form, 4'-O-[α-L-Rhamnopyranosyl- (1→6)-β-D-glucopyranoside] | Flavonoid glycoside | 37,31 | 0,00 | 5,00 | 4,07 | 2,75 | 1,00 | 6,38 | 1,78 | 61,12 | 2,05 | **2,25** | **1,14** | **0,51** | **2b** |
| 661,1382 | NI | 1,52 | -- | Flavonoid glycoside | 14,86 | 0,00 | 39,00 | 35,88 | 3,59 | 1,00 | 2,95 | 1,26 | 48,81 | 6,08 | **1,35** | **0,30** | **0,22** | **4** |
| 251,1401 | NI | 0,50 | N-dihydrocaffeoylputrescine | Hydroxy- cinnamic acid amides | 3,04 | 16,81 | 1,00 | 2,97 | 1,48 | 1,99 | 2,43 | 4,69 | 2,77 | 3,52 | **1,41** | **0,57** | **0,40** | **5** |
| 249,1244 | NI | 0,61 | N-Caffeoylputrescine | Hydroxy- cinnamic acid amides | 18,79 | 41,03 | 4,41 | 12,29 | 7,60 | 8,54 | 1,00 | 12,83 | 12,32 | 16,04 | **0,65** | **0,70** | **1,08** | **2b** |
| 529,3036 | NI | 0,76 | N1,N12-bis(dihydrocaffeoyl)  spermine | Hydroxy- cinnamic acid amides | 2,37 | 2,22 | 1,00 | 1,52 | 3,21 | 2,16 | 7,68 | 5,90 | 2,27 | 2,21 | **2,27** | **2,06** | **0,91** | **1** |
| 527,2879 | NI | 0,77 | N-(caffeoyl, dihydrocaffeoyl)  spermine | Hydroxy- cinnamic acid amides | 1,42 | 1,38 | 1,00 | 1,63 | 4,81 | 3,17 | 3,66 | 3,33 | 3,37 | 3,29 | **1,46** | **1,61** | **1,10** | **3** |
| 693,3515 | NI | 1,39 | N1,N4,N12-tris(dihydrocaffeoyl) spermine | Hydroxy- cinnamic acid amides | 20,05 | 19,65 | 6,77 | 30,63 | 1,61 | 1,00 | 168,73 | 118,16 | 3,17 | 2,19 | **9,07** | **3,52** | **0,39** | **3/4** |
| 693,3511 | NI | 1,54 | N1,N9,N12-tris(dihydrocaffeoyl)  spermine | Hydroxy- cinnamic acid amides | 20,77 | 18,50 | 17,57 | 25,21 | 3,70 | 1,00 | 79,30 | 61,69 | 1,03 | 1,51 | **2,87** | **2,12** | **0,74** | **3/4** |
| 691,3356 | NI | 1,41 | Tris(N1-caffeoyl,N4,N12-  dihydrocaffeoyl) spermine | Hydroxy- cinnamic acid amides | 6,07 | 5,59 | 6,17 | 11,33 | 2,81 | 2,19 | 21,35 | 17,35 | 1,38 | 1,00 | **2,27** | **1,44** | **0,64** | **3** |
| 857,3992 | NI | 1,91 | N1,N4,N9,N12-tetra (dihydrocaffeoyl) spermine | Hydroxy- cinnamic acid amides | 4,02 | 2,58 | 35,93 | 37,98 | 7,99 | 6,61 | 1,59 | 1,00 | nd | nd | **0,05** | **0,03** | **0,64** | **3/4** |
| 472,2456 | NI | 1,02 | N1,N8-bis(dihydrocaffeoyl)  spermidine | Hydroxy- cinnamic acid amides | 18,48 | 19,97 | 1,00 | 2,24 | 1,84 | 1,25 | 14,38 | 9,68 | 30,83 | 24,71 | **3,18** | **2,20** | **0,69** | **2b** |
| 470,2301 | NI | 1,10 | bis(N1-caffeoyl, N8-  dihydrocaffeoyl) spermidine | Hydroxy- cinnamic acid amides | 10,12 | 11,99 | 1,01 | 2,13 | 1,54 | 1,00 | 6,97 | 5,17 | 39,08 | 25,09 | **5,45** | **3,00** | **0,55** | **2b** |
| 636,2938 | NI | 1,69 | N1,N4,N8-tris(dihydrocaffeoyl)  spermidine | Hydroxy- cinnamic acid amides | 46,41 | 29,17 | 105,33 | 128,86 | 151,50 | 103,31 | 1,01 | 1,00 | 1,27 | 1,15 | **0,01** | **0,01** | **1,09** | **3/4** |
| 328,1193 | NI | 1,49 | N-feruloyl- octopamine | Hydroxy- cinnamic acid amides | 1,21 | 1,00 | 7,63 | 3,10 | 2,53 | 1,95 | 4,91 | 4,80 | 7,13 | 6,10 | **1,59** | **2,70** | **1,70** | **2b** |
| 328,1194 | NI | 1,66 | N-feruloyl- octopamine | Hydroxy- cinnamic acid amides | 1,08 | 1,00 | 2,41 | 1,51 | 1,34 | 1,18 | 2,20 | 2,03 | 2,83 | 2,44 | **1,56** | **1,82** | **1,16** | **2b** |
| 328,1194 | NI | 1,38 | N-feruloyl- octopamine | Hydroxy- cinnamic acid amides | 3,57 | 1,00 | 19,09 | 5,97 | 5,25 | 2,66 | 65,40 | 29,83 | 69,89 | 22,19 | **7,27** | **8,10** | **1,11** | **2b** |
| 312,1246 | NI | 1,97 | N-Feruloylt yramine | Hydroxy- cinnamic acid amides | 1,33 | 2,05 | 1,82 | 1,18 | 1,00 | 1,24 | 3,18 | 3,44 | 3,48 | 3,01 | **2,41** | **2,17** | **0,90** | **1** |
| 312,1246 | NI | 1,86 | N-Feruloylt yramine | Hydroxy- cinnamic acid amides | 1,67 | 1,00 | 4,80 | 5,12 | 3,90 | 2,66 | 46,46 | 16,72 | 65,48 | 14,33 | **16,19** | **5,31** | **0,33** | **2a/ 2b** |
| 623,2407 | NI | 2,76 | Grossamide | Hydroxy- cinnamic acid amides | 1,00 | 2,88 | 2,87 | 2,63 | 2,94 | 2,83 | 1,27 | 3,12 | 2,40 | 3,50 | **0,81** | **1,19** | **1,48** | **2b** |
| 623,2404 | NI | 2,58 | Grossamide | Hydroxy- cinnamic acid amides | 1,00 | 1,77 | 2,17 | 1,76 | 3,34 | 2,29 | 1,54 | 2,68 | 4,16 | 2,95 | **1,31** | **1,45** | **1,10** | **2b** |
| 282,1139 | NI | 1,88 | p-Coumaroyl- tyramine | Hydroxy- cinnamic acid amides | 6,06 | 1,00 | 15,98 | 3,83 | 3,01 | 3,04 | 2,92 | 2,65 | 4,33 | 3,33 | **0,43** | **1,14** | **2,62** | **2b** |
| 341,0882 | NI | 0,83 | caffeoyl-glucose | Hydroxy- cinnamic acids | 2,74 | 2,94 | 1,14 | 1,66 | 1,26 | 1,10 | 1,44 | 1,36 | 1,39 | 1,00 | **0,83** | **0,62** | **0,75** | **2b** |
| 163,0392 | NI | 1,24 | p-coumaric acid | Hydroxy- cinnamic acids | 6,10 | 12,69 | 31,09 | 2,07 | 1,00 | 22,35 | 6,30 | 1,21 | 9,20 | 7,16 | **0,61** | **0,34** | **0,56** | **1** |
| 163,0392 | NI | 1,33 | p-coumaric acid | Hydroxy- cinnamic acids | 52,32 | 16,04 | 71,06 | 26,27 | 1,00 | 33,43 | 4,18 | 9,09 | 25,96 | 11,36 | **0,36** | **0,40** | **1,11** | **2b** |
| 179,0342 | NI | 0,97 | Caffeic acid | Hydroxy- cinnamic acids | 1,49 | 1,26 | 1,00 | 1,46 | 1,54 | 1,63 | 2,04 | 2,09 | 1,97 | 1,90 | **1,49** | **1,38** | **0,92** | **1** |
| 337,0933 | NI | 1,08 | 4-coumaroyl  quinic acid | Hydroxy- cinnamic acids | 4,02 | 2,53 | 1,00 | 1,17 | 1,64 | 1,67 | 2,07 | 1,44 | 1,30 | 1,25 | **0,76** | **0,75** | **0,99** | **2b** |
| 335,0777 | NI | 1,28 | 4-O-caffeoyl- shikimic acid | Hydroxy- cinnamic acids | 1,64 | 1,23 | 1,00 | 1,60 | 1,09 | 1,25 | 1,45 | 1,42 | 1,95 | 1,57 | **1,37** | **1,10** | **0,80** | **2b** |
| 335,0778 | NI | 1,11 | 3-O-caffeoyl- shikimic acid | Hydroxy- cinnamic acids | 3,73 | 2,93 | 1,00 | 1,38 | 1,36 | 1,58 | 6,23 | 1,73 | 1,36 | 1,33 | **1,87** | **0,78** | **0,42** | **2b** |
| 335,0777 | NI | 1,22 | 5-O-caffeoyl- shikimic acid | Hydroxy- cinnamic acids | 1,71 | 1,54 | 1,00 | 1,51 | 1,42 | 1,62 | 1,77 | 1,62 | 2,26 | 1,79 | **1,46** | **1,09** | **0,75** | **2b** |
| 353,0881 | NI | 0,90 | 4-O-caffeoyl  quinic acid | Hydroxy- cinnamic acids | 1,49 | 1,44 | 1,00 | 1,33 | 1,23 | 1,27 | 1,52 | 1,59 | 1,50 | 1,41 | **1,22** | **1,12** | **0,92** | **1** |
| 707,1838 | NI | 0,89 | 4-O-caffeoyl  quinic acid dimer | Hydroxy- cinnamic acids | 2,59 | 2,69 | 1,00 | 2,04 | 1,44 | 1,65 | 3,62 | 3,78 | 2,55 | 2,58 | **1,84** | **1,49** | **0,81** | **1** |
| 353,0880 | NI | 0,67 | 3-O-caffeoyl  quinic acid | Hydroxy- cinnamic acids | 1,63 | 1,51 | 1,00 | 1,41 | 1,48 | 1,55 | 1,53 | 1,45 | 1,79 | 1,42 | **1,21** | **0,96** | **0,79** | **5** |
| 707,1838 | NI | 0,67 | 3-O-caffeoyl  quinic acid dimer | Hydroxy- cinnamic acids | 2,67 | 2,37 | 1,00 | 1,74 | 2,20 | 2,35 | 2,35 | 2,07 | 3,14 | 2,21 | **1,40** | **0,99** | **0,71** | **4** |
| 353,0880 | NI | 0,86 | 5-O-caffeoyl  quinic acid | Hydroxy- cinnamic acids | 1,30 | 1,33 | 1,00 | 1,25 | 1,08 | 1,18 | 1,42 | 1,47 | 1,27 | 1,35 | **1,19** | **1,13** | **0,95** | **2a/ 2b** |
| 707,1838 | NI | 0,86 | 5-O-caffeoyl  quinic acid dimer | Hydroxy- cinnamic acids | 2,41 | 2,48 | 1,00 | 2,09 | 1,16 | 1,58 | 3,14 | 3,57 | 2,11 | 2,30 | **1,72** | **1,43** | **0,83** | **4** |
| 515,1199 | NI | 1,68 | 3,5-Dicaffeoyl quinic acid | Hydroxy- cinnamic acids | 12,73 | 18,04 | 1,00 | 1,59 | 2,56 | 2,77 | 2,91 | 2,90 | 4,92 | 3,65 | **0,72** | **0,44** | **0,61** | **2b** |
| 515,1199 | NI | 1,56 | 3,4-Dicaffeoyl quinic acid | Hydroxy- cinnamic acids | 104,49 | 133,18 | 1,00 | 1,15 | 2,23 | 2,15 | 1,68 | 1,50 | 2,71 | 1,97 | **0,06** | **0,04** | **0,62** | **2b** |
| 515,1199 | NI | 1,74 | 4,5-Dicaffeoyl quinic acid | Hydroxy- cinnamic acids | 16,67 | 19,01 | 1,00 | 2,04 | 2,57 | 2,61 | 2,80 | 3,64 | 4,53 | 4,03 | **0,54** | **0,49** | **0,90** | **2b** |
| 193,0499 | NI | 1,46 | Ferulic acid | Hydroxy- cinnamic acids | 37,38 | 31,60 | 15,48 | 22,46 | 14,57 | 13,29 | 45,60 | 42,82 | 1,25 | 1,00 | **1,04** | **0,98** | **0,94** | **2a/ 2b** |
| 367,1038 | NI | 1,19 | 5-O-Feruloyl quinic acid | Hydroxy- cinnamic acids | 3,65 | 2,52 | 1,51 | 1,00 | 2,55 | 2,70 | 2,89 | 3,06 | 4,31 | 3,50 | **1,40** | **1,58** | **1,13** | **2a/ 2b** |
| 367,1038 | NI | 0,94 | 3-O-Feruloyl quinic acid | Hydroxy- cinnamic acids | 3,00 | 2,24 | 2,19 | 1,18 | 4,85 | 5,19 | 1,02 | 1,00 | 2,21 | 1,20 | **0,48** | **0,38** | **0,79** | **2b** |
| 367,1038 | NI | 1,32 | 4-O-Feruloyl quinic acid | Hydroxy- cinnamic acids | 1,98 | 2,87 | 1,06 | 2,65 | 1,00 | 1,56 | 2,40 | 4,05 | 1,89 | 2,87 | **1,59** | **1,46** | **0,92** | **2b** |
| 223,0610 | NI | 1,41 | Sinapic acid | Hydroxy- cinnamic acids | 2,12 | 2,87 | 1,00 | 1,86 | 2,55 | 2,39 | 6,60 | 5,91 | 2,13 | 2,22 | **2,31** | **1,71** | **0,74** | **2a/ 4** |
| 385,1145 | NI | 1,05 | Sinapoyl malate | Hydroxy- cinnamic acids | 19,25 | 19,78 | 1,00 | 4,45 | 4,95 | 6,55 | 48,01 | 41,70 | 1,69 | 1,24 | **2,96** | **2,09** | **0,71** | **2a/ 4** |
| 339,0724 | NI | 1,47 | Sinapoyl  glucoside | Hydroxy- cinnamic acids | 4,09 | 9,50 | 1,00 | 1,96 | 2,05 | 2,46 | 2,77 | 1,89 | 1,84 | 2,09 | **0,97** | **0,43** | **0,44** | **2a/ 4** |
| 575,3570 | PI | 2,13 | 24,25-Epoxy-7,22- dihydroxyergosta-1,4-dien-3-one | Steroid  derivative | 2,49 | 1,80 | 1,00 | 1,08 | 1,57 | 1,35 | 4,04 | 3,79 | 5,13 | 4,46 | **2,73** | **2,92** | **1,07** | **2b/ 4** |
| 883,4662 | PI | 2,46 | 3,23-Dihydroxy-30-nor-12,20(29) -oleanadien-28-oic acid;  3β-form, 3-O-[β-D-Xylopyranosyl-(1→2)-[β-D-glucopyranosyl-(1→3)]-α-L-arabinopyranoside] | Steroid  derivative | 1,49 | 1,00 | 5,35 | 4,02 | 2,24 | 1,92 | 5,78 | 4,16 | 10,71 | 10,61 | **2,72** | **3,19** | **1,17** | **2b/ 4** |
| 883,4665 | PI | 2,11 | 3,23-Dihydroxy-30-nor-12,20(29)- oleanadien-28-oic acid; 3β-form, 3-O-[β-D-Xylopyranosyl-(1→2)-[β-D-glucopyranosyl-(1→3)]-α-L-arabinopyranoside] | Steroid  derivative | 9,77 | 7,17 | 1,55 | 1,00 | 35,98 | 33,14 | 109,07 | 74,22 | 4,87 | 4,53 | **3,61** | **2,86** | **0,79** | **2b/ 4** |
| 883,4700 | PI | 1,85 | 3,23-Dihydroxy-30-nor-12,20(29)- oleanadien-28-oic acid; 3β-form, 3-O-[β-D-Xylopyranosyl-(1→2)-[β-D-glucopyranosyl-(1→3)]-α-L-arabinopyranoside] | Steroid  derivative | 8,68 | 5,90 | 2,53 | 1,00 | 51,63 | 39,87 | 85,46 | 64,54 | nd | nd | **2,04** | **2,07** | **1,01** | **2b/ 4** |
| 398,3413 | PI | 2,75 | Solanidine | Steroidal  alkaloid | 2,53 | 19,26 | 1,00 | 8,80 | 1,93 | 8,27 | 1,88 | 6,59 | 2,47 | 9,89 | **1,19** | **0,68** | **0,57** | **1** |
| 398,3413 | PI | 2,14 | Solanidine | Steroidal  alkaloid | 3,22 | 2,46 | 1,20 | 1,00 | 2,52 | 2,11 | 2,71 | 2,57 | 3,04 | 2,62 | **1,24** | **1,40** | **1,13** | **2b** |
| 400,3560 | PI | 2,01 | Demissidine | Steroidal  alkaloid | 4,69 | 2,45 | 164,00 | 214,67 | 2,04 | 1,00 | 69,73 | 82,00 | 145,33 | 248,00 | **1,90** | **2,27** | **1,20** | **2b/ 4** |
| 414,3366 | PI | 1,99 | Solasodine | Steroidal  alkaloid | 1,59 | 1,00 | 10,36 | 9,36 | 7,37 | 4,89 | 2,32 | 1,61 | 2,45 | 1,32 | **0,37** | **0,29** | **0,78** | **2a/ 2b/ 4** |
| 706,4516 | PI | 2,16 | beta-chaconine | Steroidal  alkaloid | 1,88 | 1,70 | 1,00 | 1,06 | 1,54 | 1,39 | 1,95 | 2,00 | 2,25 | 1,99 | **1,43** | **1,44** | **1,01** | **2b** |
| 721,4155 | PI | 2,49 | 16,23:16,24-Diepoxycycloartane- 3,12,15,25-tetrol; (3β,12β,15α,16S,23R,24S)-form, 12-Ac, 3-O-(3-O-acetyl-β-D-xylopyranoside) | Steroidal  glycoside | 1,86 | 1,00 | 5,33 | 5,56 | 3,28 | 2,60 | 8,97 | 7,46 | 24,59 | 22,54 | **4,81** | **4,92** | **1,02** | **2b/ 4** |
| 750,4443 | NI | 2,20 | Solanidine;  O-[α-L-Rhamnopyranosyl-(1→4)-β-D-glucopyranoside] | Steroidal  glycoside | 2,42 | 3,48 | 1,37 | 2,62 | 1,00 | 1,66 | 1,33 | 1,77 | 4,65 | 6,14 | **1,87** | **1,53** | **0,82** | **2b/ 4** |
| 986,4983 | NI | 2,37 | 3,16-Dihydroxypregn-5-en-20-one;  (3β,16β)-form, 16-O-(2-Methoxy-4-methylpyrrolidine-2-carbonyl), 3-O-[α-L-rhamnopyranosyl-(1→2)-[α-L-rhamnopyranosyl-(1→4)]-β-D-glucopyranoside] | Steroidal  glycoside | 9,90 | 4,70 | 1,71 | 1,00 | 2,51 | 3,35 | nd | nd | 30,07 | 20,20 | **3,19** | **3,35** | **1,05** | **2b/ 4** |
| 771,3459 | PI | 1,95 | -- | Steroidal  glycoside | nd | nd | 1,02 | 1,00 | nd | nd | nd | nd | 1,97 | 1,90 | **2,41** | **2,54** | **1,05** | **4** |
| 914,4388 | PI | 2,26 | -- | Steroidal  glycoside | nd | nd | 1,00 | 1,01 | nd | nd | nd | nd | 1,87 | 1,71 | **2,80** | **2,54** | **0,91** | **4** |
| 918,4147 | PI | 2,63 | -- | Steroidal  glycoside | nd | nd | nd | nd | nd | nd | 1,35 | 1,00 | nd | nd | **∞** | **∞** | **∞** | **4** |
| 966,4570 | PI | 2,00 | -- | Steroidal  glycoside | nd | nd | nd | nd | 2,19 | 1,00 | 85,71 | 87,58 | nd | nd | **59,03** | **131,57** | **2,23** | **4** |
| 987,4357 | PI | 2,61 | -- | Steroidal  glycoside | nd | nd | 1,00 | 1,18 | nd | nd | nd | nd | 1,27 | 1,11 | **1,90** | **1,41** | **0,74** | **4** |
| 1049,4836 | PI | 1,84 | -- | Steroidal  glycoside | nd | nd | 1,02 | 1,00 | nd | nd | nd | nd | 1,97 | 1,90 | **2,89** | **2,86** | **0,99** | **4** |
| 1065,5651 | PI | 2,14 | -- | Steroidal  glycoside | 1,13 | 1,06 | 1,41 | 1,86 | 1,14 | 1,00 | 1,85 | 2,24 | 5,42 | 5,50 | **2,96** | **2,97** | **1,00** | **4** |
| 1196,6059 | PI | 2,03 | -- | Steroidal  glycoside | 2,17 | 1,00 | 12,09 | 3,87 | 45,40 | 38,34 | 322,09 | 227,30 | 40,18 | 48,77 | **9,08** | **9,57** | **1,05** | **4** |
| 577,3737 | PI | 2,69 | 6-Hydroxyspirostan-3-one;  (5α,6α,25S)-form, 6-O-α-L-Rhamnopyranoside | Steroidal  Saponin | 2,13 | 1,00 | 8,30 | 6,12 | 1,68 | 1,65 | 7,16 | 4,21 | 4,13 | 3,13 | **1,40** | **1,25** | **0,90** | **2b/ 4** |
| 577,3738 | PI | 2,87 | 6-Hydroxyspirostan-3-one;  (5α,6α,25S)-form, 6-O-α-L-Rhamnopyranoside | Steroidal  Saponin | 2,10 | 1,00 | 2,17 | 1,84 | 3,82 | 1,55 | 3,09 | 1,91 | 3,42 | 2,19 | **1,21** | **1,40** | **1,16** | **2b/ 4** |
| 721,4158 | PI | 2,13 | 16,23:16,24-Diepoxycycloartane -3,12,15,25-tetrol; (3β,12β,15α,16S,23R,24S)-form, 12-Ac, 3-O-(3-O-acetyl-β-D-xylopyranoside) | Steroidal  Saponin | 2,24 | 1,65 | 1,00 | 1,17 | 1,50 | 1,35 | 4,49 | 4,12 | 5,57 | 5,19 | **3,19** | **3,35** | **1,05** | **2b/ 4** |
| 737,4104 | PI | 2,11 | Spirosta-5,25(27)-diene-1,3-diol; (1β,3β) -form, 3-O-[α-L-Rhamnopyranosyl-(1→4)-β-D-glucopyranoside] | Steroidal  Saponin | 5,69 | 3,48 | 1,32 | 1,00 | 15,56 | 13,24 | 45,74 | 31,39 | 1,99 | 1,83 | **3,17** | **2,81** | **0,89** | **2b/ 4** |
| 867,4721 | PI | 2,50 | Spirosta-5,25(27)-dien-3-ol;  3β-form, 3-O-[α-L-Rhamnopyranosyl-(1→2)-[α-L-rhamnopyranosyl-(1→4)]-β-D-glucopyranoside] | Steroidal  Saponin | 1,57 | 1,00 | 9,66 | 10,19 | 5,40 | 3,94 | 14,88 | 10,94 | 42,37 | 39,97 | **5,16** | **5,05** | **0,98** | **2b/ 4** |
| 867,4733 | PI | 3,60 | Spirosta-5,25(27)-dien-3-ol;  3β-form, 3-O-[α-L-Rhamnopyranosyl-(1→2)-[α-L-rhamnopyranosyl-(1→4)]-β-D-glucopyranoside] | Steroidal  Saponin | 2,01 | 1,00 | 8,41 | 7,63 | 6,31 | 5,70 | 13,52 | 8,97 | 41,96 | 37,39 | **4,97** | **4,86** | **0,98** | **2b/ 4** |
| 885,4833 | PI | 2,17 | Spirost-5-en-3-ol; (3β,25R)- form, 3-O-[α-L-Rhamnopyranosyl-(1→2)-[β-D-glucopyranosyl-(1→3)]-β-D-galactopyranoside] | Steroidal  Saponin | 2,31 | 1,00 | 5,78 | 5,02 | 2,60 | 1,88 | 7,15 | 3,88 | 1,70 | 1,21 | **1,24** | **0,97** | **0,78** | **2b/ 4** |
| 896,4982 | NI | 2,11 | Solanidine;  O-[α-L-Rhamnopyranosyl-(1→2)-O-[α-L-rhamnopyranosyl-(1→4)]-β-D-glucopyanoside] | Steroidal  Saponin | 8,64 | 3,27 | 1,43 | 1,56 | 1,40 | 1,00 | 4,72 | 10,93 | 79,49 | 4,15 | **11,02** | **3,88** | **0,35** | **2b/ 4** |
| 915,4575 | PI | 2,08 | 3,15,23-Trihydroxyspirost -5-en-26-one; (3β,15α,23R,25R)-form, 3-O-[α-L-Rhamnopyranosyl-(1→2)-[α-L-rhamnopyranosyl-(1→4)]-β-D-glucopyranoside] | Steroidal  Saponin | 10,54 | 3,96 | 23,35 | 16,06 | 1,31 | 1,00 | 118,23 | 120,20 | 6,45 | 6,40 | **5,30** | **9,04** | **1,71** | **2b/ 4** |
| 929,4764 | NI | 3,60 | Spirost-5-ene-3,12-diol;  (3β,12β,25R)-form, 3-O-[α-L-Rhamnopyranosyl-(1→2)-[α-L-rhamnopyranosyl-(1→4)]-β-D-glucopyranoside] | Steroidal  Saponin | 1,47 | 1,00 | 5,91 | 4,73 | 3,63 | 3,10 | 16,93 | 18,13 | 8,46 | 5,70 | **3,46** | **4,05** | **1,17** | **2b/ 4** |
| 929,4769 | NI | 2,49 | Spirost-5-ene-3,12-diol;  (3β,12β,25R)-form, 3-O-[α-L-Rhamnopyranosyl-(1→2)-[α-L-rhamnopyranosyl-(1→4)]-β-D-glucopyranoside] | Steroidal  Saponin | 1,59 | 1,00 | 5,50 | 6,32 | 3,61 | 2,80 | 24,16 | 21,40 | 8,19 | 6,06 | **4,53** | **4,07** | **0,90** | **2b/ 4** |
| 933,5067 | NI | 2,65 | Cholestane-3,16,22,26-tetrol;  (3β,5α,16α,25ξ)-form, 22-Ketone, 3-O-[β-D-xylopyranosyl-(1→2)-α-L-rhamnopyranosyl-(1→4)-β-D-glucopyranoside] | Steroidal  Saponin | 36,95 | 26,11 | 2,70 | 1,23 | 1,84 | 1,00 | 2,76 | 2,86 | 40,24 | 30,34 | **1,55** | **1,76** | **1,13** | **2b/ 4** |
| 945,4715 | NI | 2,45 | Spirost-5-ene-3,12-diol;  (3β,12β,25R)-form, 3-O-[α-L-Rhamnopyranosyl-(1→2)-[β-D-glucopyranosyl-(1→3)]-β-D-galactopyranoside] | Steroidal  Saponin | 1,19 | 1,00 | 2,26 | 1,99 | 2,08 | 1,72 | 4,94 | 5,41 | 3,67 | 2,33 | **2,34** | **2,46** | **1,05** | **2b/ 4** |
| 945,4716 | NI | 2,11 | Spirost-5-ene-3,12,26-triol;  (3β,12β,25R,26R)-form, 3-O-[α-L-Rhamnopyranosyl-(1→2)-[α-L-rhamnopyranosyl-(1→4)]-β-D-glucopyranoside] | Steroidal  Saponin | 2,27 | 1,20 | 1,36 | 1,00 | 169,03 | 146,78 | 2,84 | 2,76 | 457,63 | 365,58 | **4,00** | **3,71** | **0,93** | **2b/ 4** |
| 948,4945 | PI | 2,40 | 26-Aminospirost-5-en-3-ol;  (3β,25R,26R)-form, N-Ac, 3-O-[α-L-rhamnopyranosyl-(1→2)-[α-L-rhamnopyranosyl-(1→4)]-β-D-glucopyranoside] | Steroidal  Saponin | 4,48 | 2,31 | 2,55 | 2,23 | 1,00 | 1,33 | 16,19 | 9,85 | nd | nd | **3,02** | **2,52** | **0,83** | **2b/ 4** |
| 949,4981 | NI | 2,20 | Furostane-3,6,22,26-tetrol;  (3β,5α,6α,22ξ,25S)-form, 6-O-[α-L-Rhamnopyranosyl-(1→3)-6-deoxy-β-D-glucopyranoside], 26-O-β-D-glucopyranoside | Steroidal  Saponin | 4,16 | 1,00 | 6,14 | 4,29 | 4,57 | 4,78 | 12,98 | 10,92 | 5,02 | 3,15 | **1,81** | **2,10** | **1,16** | **2b/ 4** |
| 949,5000 | PI | 2,40 | Furost-5-ene-1,3,22,26-tetrol;  (1β,3β,22ξ,25S)-form, 22-Me ether, 3-O-[β-D-glucopyranosyl-(1→4)-β-D-galactopyranoside], 26-O-β-D-glucopyranoside | Steroidal  Saponin | 4,72 | 1,50 | 2,56 | 2,17 | 1,00 | 1,26 | 14,72 | 6,90 | nd | nd | **2,68** | **2,11** | **0,79** | **2b/ 4** |
| 970,5031 | NI | 2,40 | 26-Aminospirost-5-en-3-ol;  (3β,25R,26R)-form, N-Ac, 3-O-[α-L-rhamnopyranosyl-(1→2)-[α-L-rhamnopyranosyl-(1→4)]-β-D-glucopyranoside] | Steroidal  Saponin | 4,03 | 1,61 | 1,73 | 1,41 | 1,00 | 1,03 | nd | nd | 13,57 | 9,61 | **3,01** | **3,55** | **1,18** | **2b/ 4** |
| 1031,5413 | PI | 2,18 | beta-D-Glucopyranoside,  (3beta,22beta,25R)-26-(beta-D-glucopyranosyloxy)-22-hydroxyfurost-5-en-3-yl O-6-deoxy-alpha-L-mannopyranosyl-(1->2)-O-[6-deoxy-alpha-L-mannopyranosyl-(1->4)]- | Steroidal  Saponin | 2,42 | 1,24 | 8,87 | 8,15 | 1,58 | 1,00 | 10,39 | 5,05 | 2,98 | 2,76 | **1,56** | **1,13** | **0,72** | **2a/ 2b/ 4** |
| 1047,5363 | PI | 1,71 | Cholest-5-ene-3,16,22,26-tetrol;  (3β,16β,22ξ,25R)-form, 16,22-Diketone, 3-O-[α-L-rhamnopyranosyl-(1→2)-[α-L-rhamnopyranosyl-(1→4)]-β-D-glucopyranoside], 26-O-β-D-glucopyranoside | Steroidal  Saponin | 131,97 | 90,16 | 39,43 | 26,48 | 118,85 | 94,26 | 511,48 | 260,66 | 2,44 | 1,00 | **2,66** | **1,87** | **0,70** | **2b/ 4** |
| 1048,5677 | PI | 2,70 | Spirosolan-3-ol; (3β,5α,22R,25R)- form, 3-O-[β-D-Glucopyranosyl-(1→2)-β-D-glucopyranosyl-(1→4)-[α-L-rhamnopyranosyl-(1→2)]-β-D-galactopyranoside] | Steroidal  Saponin | 2,43 | 1,03 | 12,89 | 10,79 | 1,13 | 1,00 | 17,32 | 8,53 | 4,86 | 3,89 | **2,03** | **1,45** | **0,72** | **2b/ 4** |
| 1064,5629 | PI | 2,13 | Leptinidine; 3-O-[β-D-Glucopyranosyl -(1→2)-[β-D-xylopyranosyl-(1→3)]-β-D-glucopyranosyl-(1→4)-β-D-galactopyranoside] | Steroidal  Saponin | 1,34 | 1,25 | 1,21 | 1,48 | 1,09 | 1,00 | 1,98 | 2,16 | 4,69 | 4,93 | **2,75** | **2,85** | **1,04** | **2b/ 4** |
| 1075,5342 | NI | 2,69 | 22,25-Epoxyfurost-5-ene-3,7,26-triol;  (3β,7β,22S,25S)-form, 7-Me ether, 3-O-[α-L-rhamnopyranosyl-(1→2)-[α-L-rhamnopyranosyl-(1→4)]-β-D-glucopyranoside], 26-O-β-D-glucopyranoside | Steroidal  Saponin | 2,52 | 1,00 | 8,18 | 9,00 | 1,23 | 1,12 | 4,70 | 3,57 | 9,18 | 6,23 | **1,74** | **1,32** | **0,76** | **2b/ 4** |
| 1079,5650 | NI | 2,25 | Cholest-5-ene-3,22,26-triol;  (3β,22R,25S)-form, 3-O-[α-L-Rhamnopyranosyl-(1→2)-[α-L-rhamnopyranosyl-(1→4)]-β-D-glucopyranoside], 26-O-β-D-glucopyranoside | Steroidal  Saponin | 191,87 | 123,68 | 5,40 | 6,04 | 1,63 | 1,00 | 8,84 | 10,00 | 200,37 | 160,85 | **1,58** | **1,96** | **1,24** | **2b/ 4** |
| 1091,5293 | NI | 2,11 | Nuatigenin;  3-O-[α-L-Rhamnopyranosyl-(1→2)-[β-D-xylopyranosyl-(1→3)]-β-D-galactopyranoside], 26-O-β-D-glucopyranoside | Steroidal  Saponin | 2,97 | 2,75 | 2,14 | 2,61 | 3,11 | 2,72 | 7,50 | 8,53 | 1,00 | 2,52 | **1,55** | **2,05** | **1,32** | **2b/ 4** |
| 1093,5438 | NI | 2,28 | Protodioscin/ neoprotodioscin | Steroidal  Saponin | 2,90 | 1,76 | 3,13 | 3,49 | 1,16 | 1,00 | 2,45 | 2,97 | 5,34 | 4,15 | **1,62** | **1,71** | **1,05** | **2b/ 4** |
| 1093,5438 | NI | 2,17 | Protodioscin/ neoprotodioscin | Steroidal  Saponin | 1,87 | 1,03 | 8,12 | 7,55 | 1,39 | 1,00 | 2,83 | 2,69 | 7,82 | 4,30 | **1,40** | **1,09** | **0,78** | **1** |
| 1107,5245 | NI | 2,10 | Methyl- protodioscin | Steroidal  Saponin | 7,07 | 5,62 | 1,64 | 1,00 | 3,60 | 2,88 | 4,02 | 3,88 | 4,11 | 4,17 | **0,99** | **1,27** | **1,28** | **2b/ 4** |
| 1107,5246 | NI | 2,27 | Methyl- protodioscin | Steroidal  Saponin | 1,97 | 1,42 | 2,13 | 1,10 | 1,35 | 1,01 | 1,06 | 1,04 | 1,14 | 1,00 | **0,61** | **0,87** | **1,43** | **2b/ 4** |
| 1109,5395 | NI | 1,71 | Spirostane-3,15-diol;  (3β,5α,15α,25R)-form, 3-O-[β-D-Glucopyranosyl-(1→2)-[β-D-xylopyranosyl-(1→3)]-β-D-glucopyranosyl-(1→4)-β-D-galactopyranoside] | Steroidal  Saponin | 41,47 | 28,90 | 8,47 | 6,23 | 28,02 | 22,68 | 1,00 | 1,11 | 113,88 | 74,30 | **2,21** | **1,96** | **0,89** | **2b/ 4** |
| 1223,5720 | NI | 2,04 | Spirost-5-ene-3,27-diol;  (3β,25S)-form, 3-O-[β-D-Xylopyranosyl-(1→2)-α-L-rhamnopyranosyl-(1→4)-[α-L-rhamnopyranosyl-(1→2)]-β-D-glucopyranoside], 27-O-β-D-glucopyranoside | Steroidal  Saponin | 1,40 | 1,00 | 5,91 | 2,55 | 21,93 | 19,13 | 20,71 | 25,16 | 139,26 | 112,01 | **8,21** | **9,08** | **1,11** | **2b/ 4** |
| 722,4470 | PI | 2,14 | beta-solanine | Steroidal  Saponin | 4,27 | 3,36 | 1,41 | 1,00 | 3,22 | 2,73 | 3,16 | 3,12 | 3,59 | 3,54 | **1,14** | **1,41** | **1,24** | **2b** |
| 852,5091 | PI | 2,16 | alpha-chaconine | Steroidal  Saponin | 1,94 | 1,67 | 1,11 | 1,00 | 1,62 | 1,42 | 2,13 | 2,16 | 2,21 | 1,87 | **1,39** | **1,48** | **1,06** | **1** |
| 866,4891 | PI | 1,90 | 7-Hydroxysolanidine;  (3β,7β)-form, 7-Ketone, 3-O-[α-L-rhamnopyranosyl-(1→2)-[α-L-rhamnopyranosyl-(1→4)]-β-D-glucopyranoside] | Steroidal  Saponin | 2,97 | 1,86 | 1,56 | 1,00 | 2,52 | 2,06 | 3,33 | 3,71 | 5,24 | 3,55 | **1,82** | **2,21** | **1,21** | **2b/ 4** |
| 868,5038 | PI | 2,14 | alpha-solanine | Steroidal  Saponin | 3,40 | 2,89 | 1,35 | 1,00 | 2,76 | 2,39 | 2,55 | 2,69 | 3,28 | 2,87 | **1,16** | **1,33** | **1,14** | **1** |
| 868,5043 | PI | 1,55 | alpha-solanine  isomer | Steroidal  Saponin | 1,44 | 1,00 | 3,95 | 1,47 | 8,38 | 7,35 | 1,46 | 1,19 | 17,36 | 12,10 | **2,05** | **2,03** | **0,99** | **2b** |
| 882,4846 | PI | 1,89 | 7-Hydroxysolanidine;  (3β,7β)-form, 7-Ketone, 3-O-[α-L-rhamnopyranosyl-(1→2)-[β-D-glucopyranosyl-(1→3)]-β-D-galactopyranoside] | Steroidal  Saponin | 3,76 | 2,36 | 1,35 | 1,00 | 3,22 | 2,86 | 2,69 | 2,96 | 5,71 | 4,04 | **1,51** | **1,69** | **1,12** | **2b/ 4** |
| 884,4993 | PI | 1,97 | Spirosol-5-en-3-ol;  (3β,16β,22S,25S)-form, 3-O-[α-L-Rhamnopyranosyl-(1→2)-β-D-glucopyranosyl-(1→3)-β-D-galactopyranoside] | Steroidal  Saponin | 1,57 | 1,00 | 6,08 | 4,45 | 6,06 | 3,72 | 1,81 | 1,53 | 2,24 | 1,33 | **0,44** | **0,47** | **1,05** | **2b/ 4** |
| 910,5153 | PI | 2,49 | Leptinidine;  23-Ac, 3-O-[α-L-rhamnopyranosyl-(1→2)-[α-L-rhamnopyranosyl-(1→4)]-β-D-glucopyranoside] | Steroidal  Saponin | 4,53 | 3,43 | 1,25 | 1,00 | 3,18 | 2,78 | 3,30 | 3,77 | 4,90 | 4,48 | **1,37** | **1,71** | **1,25** | **2b** |
| 910,5158 | PI | 2,08 | Leptinidine;  23-Ac, 3-O-[α-L-rhamnopyranosyl-(1→2)-[α-L-rhamnopyranosyl-(1→4)]-β-D-glucopyranoside] | Steroidal  Saponin | 24,59 | 17,87 | 4,23 | 2,70 | 2,87 | 3,89 | 36,11 | 32,03 | 1,19 | 1,00 | **1,77** | **2,03** | **1,15** | **2b** |
| 547,2403 | NI | 1,56 | 9,13-Dihydroxy- 4,7-megastigmadien-3-one; (6R,7E,9R)-form, 9,13-Di-O-β-D-glucopyranoside | Terpene  glycoside | 2,56 | 4,17 | 3,57 | 4,51 | 2,03 | 3,65 | 1,09 | 1,00 | 9,35 | 8,81 | **1,92** | **1,19** | **0,62** | **2b/ 4** |
| 160,1078 | PI | 0,39 | Calystegine A3 | Tropane  alkaloid | 2,81 | 2,29 | nd | 4,50 | 3,00 | 3,25 | 2,61 | 1,00 | 3,44 | 3,14 | **2,07** | **1,54** | **0,74** | **1** |
| 176,0917 | PI | 0,25 | Calystegine B2 | Tropane  alkaloid | 1,51 | 2,65 | 3,82 | 6,82 | 5,35 | 1,00 | 2,67 | 4,05 | 1,00 | 1,90 | **1,40** | **1,16** | **0,83** | **2b/ 4** |
| 179,0340 | PI | 0,96 | Esculetin | Hydroxy- coumarin | 4,19 | 1,00 | 4,02 | 3,17 | 2,44 | 1,63 | 20,48 | 6,47 | 17,44 | 6,24 | **5,34** | **3,29** | **0,62** | **1** |
| 191,0342 | NI | 1,38 | Scopoletin | Hydroxy- coumarin | 4,13 | 11,93 | 24,68 | 13,58 | 1,51 | 1,00 | 1,80 | 1,96 | 6,59 | 17,67 | **0,42** | **1,12** | **2,39** | **1** |
| 223,0601 | PI | 1,46 | Isofraxidin | Hydroxy- coumarin | 1,00 | 15,23 | 7,58 | 32,22 | 1,06 | 4,64 | 1,50 | 10,75 | 1,34 | 32,47 | **0,44** | **1,24** | **2,81** | **2a** |
| 223,0602 | PI | 0,99 | Isofraxidin | Hydroxy- coumarin | 1,82 | 88,75 | 24,52 | 170,21 | 5,87 | 17,93 | 1,00 | 34,08 | 2,32 | 150,96 | **0,15** | **1,18** | **6,31** | **2a** |
